# Covalent bond shortening and distortion induced by pressurization of thorium, uranium, and neptunium tetrakis aryloxides

**DOI:** 10.1038/s41467-022-33459-7

**Published:** 2022-10-07

**Authors:** Jacob J. Shephard, Victoria E. J. Berryman, Tatsumi Ochiai, Olaf Walter, Amy N. Price, Mark R. Warren, Polly L. Arnold, Nikolas Kaltsoyannis, Simon Parsons

**Affiliations:** 1 EaStCHEM School of Chemistry and The Centre for Science at Extreme Conditions, The University of Edinburgh, King’s Buildings, West Mains Road, Edinburgh, EH9 3FJ UK; 2 Department of Chemistry, The University of Manchester, Oxford Road, Manchester, M13 9PL UK; 3 Lawrence Berkeley National Laboratory, University of California, Berkeley, Berkeley, CA CA94704 USA; 4 European Commission Joint Research Centre, P.O. Box 2340, 76125 Karlsruhe, Germany; 5 Diamond Light Source, Harwell Science and Innovation Campus, Didcot, Oxfordshire, OX11 0DE UK

**Keywords:** Chemical bonding, Coordination chemistry

## Abstract

Covalency involving the 5f orbitals is regularly invoked to explain the reactivity, structure and spectroscopic properties of the actinides, but the ionic versus covalent nature of metal-ligand bonding in actinide complexes remains controversial. The tetrakis 2,6-di-*tert*-butylphenoxide complexes of Th, U and Np form an isostructural series of crystal structures containing approximately tetrahedral MO_4_ cores. We show that up to 3 GPa the Th and U crystal structures show negative linear compressibility as the OMO angles distort. At 3 GPa the angles snap back to their original values, reverting to a tetrahedral geometry with an abrupt shortening of the M-O distances by up to 0.1 Å. The Np complex shows similar but smaller effects, transforming above 2.4 GPa. Electronic structure calculations associate the M-O bond shortening with a change in covalency resulting from increased contributions to the M-O bonding by the metal 6d and 5f orbitals, the combination promoting MO_4_ flexibility at little cost in energy.

## Abstract (web summary)

The properties of actinide complexes depend on the ionic versus covalent nature of the bonds. Here, the authors report that pressure can distort actinide-oxygen bonding; differences seen for Th, U and Np result from the decreasing size of the atoms.

## Introduction

High pressure has become established as a sub-discipline of crystal engineering, where it is used to characterise and modify intermolecular interactions and promote polymorphism. Its application in controlling intramolecular interactions is rarer. This is particularly true in organic solids, where covalent bonds are generally robust towards pressures up to 10 GPa^1^. In the d-block, changes in internal Mn-N-O-Mn torsion angles in the [MnF_6_] family of single molecule magnets interconvert ferro- and antiferro-magnetic coupling between the Mn centres^2^.

Within the f-block, pressure can be used to modify the structures and bonding in actinide complexes. The relative influence of steric effects, agostic bonding, dipole moments and volume in determining the pyramidal versus planar structures of trivalent uranium complexes has been revealed recently at pressures up to 5 GPa^3^. Similar pressures applied to the amido inverse arene sandwich [N“_2_U]_2_(μ-C_6_H_6_) (N” = N(SiMe_3_)_2_) result in a shortening of the U–U distance from 4.2492(2) Å to 4.191(5) Å with the formation of agostic U···H bonds, evidenced by Quantum Theory of Atoms in Molecules (QTAIM) and Natural Bond Orbital (NBO) calculations^4^. High-pressure optical spectroscopy and supporting DFT calculations on the 5f^7^ actinide complex [Cm(pydtc)_4_]^−^ (pydtc = S_2_C(*cy-*NC_4_H_8_), pyrrolidinedithiocarbamate), the related O-bonded Cm_2_(mel)(OH_2_)_8_.2H_2_O (mel = [C_6_(CO_2_)_6_]^6−^, mellitate), and [Nd(pydtc)_4_]^−^ (4f^2^) have shown that the Cm-S bonds are most affected by pressure, with the Cm contribution to the Cm-S bonds increased from 8 to 13% by 11 GPa, and the fraction of 5f-orbital contribution to the bond also increased from 10 to 19%^5^.

Our aim in this paper is to show how pressure influences the ionic versus covalent nature of bonding in complexes of early 5f metals. While the core-like nature of 4f-orbitals in the lanthanides is the most important feature of the electronic structures of complexes of these metals, the same is not true for the 5f orbitals in the early and middle actinides. Small but significant covalency involving the 5f orbitals is regularly invoked to explain the reactivity, structure and spectroscopic properties of these elements^6,7^, and has been probed with spectroscopic^8–10^ and computational methods^11–13^. QTAIM, which is based on topological analysis of the electron density, has proved particularly useful in distinguishing overlap- from energy-driven covalency in the actinide series^14^.

In the isolated actinide atoms, the energies of the 5f orbitals, and to a lesser extent the 6d, drop on crossing the actinide series, but reliable prediction of the d- and f-orbital energies in a complex as a function of ligand and metal identity and oxidation state remains elusive. Understanding the differences in electronic structure and orbital covalency is fundamentally important for the segregation and manipulation of the f-block ions in technological and energy applications. The differences in orbital involvement also have structural implications, e.g. Th forms *cis-*geometry bis(imido) complexes^15^, like the d^0^ transition metal analogues, but the involvement of the 5f orbitals results in linearity in all the d^0^f^0^ early actinide (U, Np, Pu, Am) bis(oxo) compounds and 59 of the 70 known uranium bis(imido) complexes^16,17^.

**Fig. 1:**
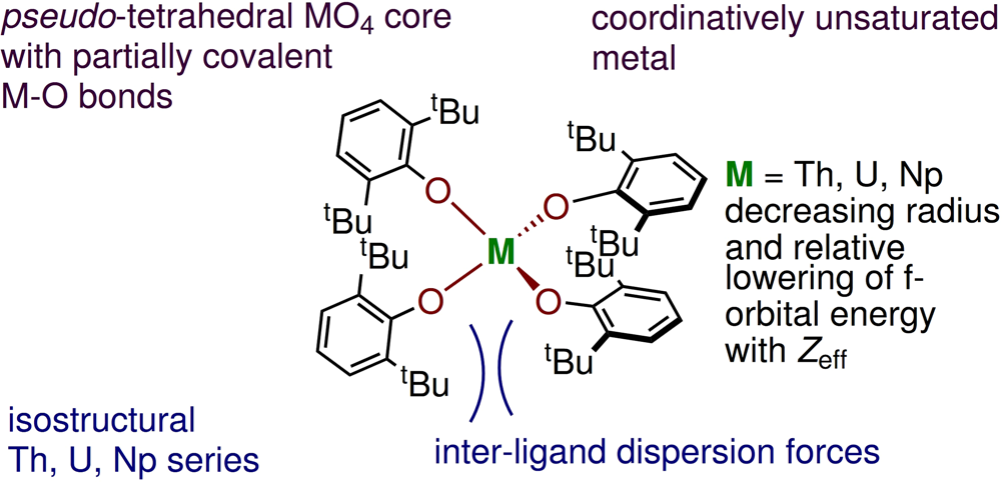
Pseudo tetrahedral actinide molecules investigated in this study. Chemical structure of tetra-coordinated early actinide aryloxides M(OAr)_4_ (M = Th, U, Np; OAr = 2,6-di-*tert*-butylphenoxide).

We describe below the effects of pressure on three crystallographically isostructural tetra-coordinated early actinide aryloxides M(OAr)_4_ (M = Th, U, Np; OAr = 2,6-di-*tert*-butylphenoxide) (Fig. 1), with a combination of single crystal X-ray diffraction and DFT calculations. This simple series has been chosen to highlight differences between the three metals, demonstrating the extent to which the degree of covalency can be manipulated by externally-applied conditions.

## Results

### The crystal structures of M(OAr)_4_ at high pressure

The uranium and thorium aryloxides were prepared by modifications of literature procedures^18–20^. The neptunium (IV) complex Np(OAr)_4_ was synthesised by reaction of NpCl_4_ with KOAr, analogous to the syntheses of U(OAr)_4_ and Th(OAr)_4_. Details of these reactions and those to target the smaller Zr and Ce analogues are included in the Supplementary Information.

**Fig. 2:**
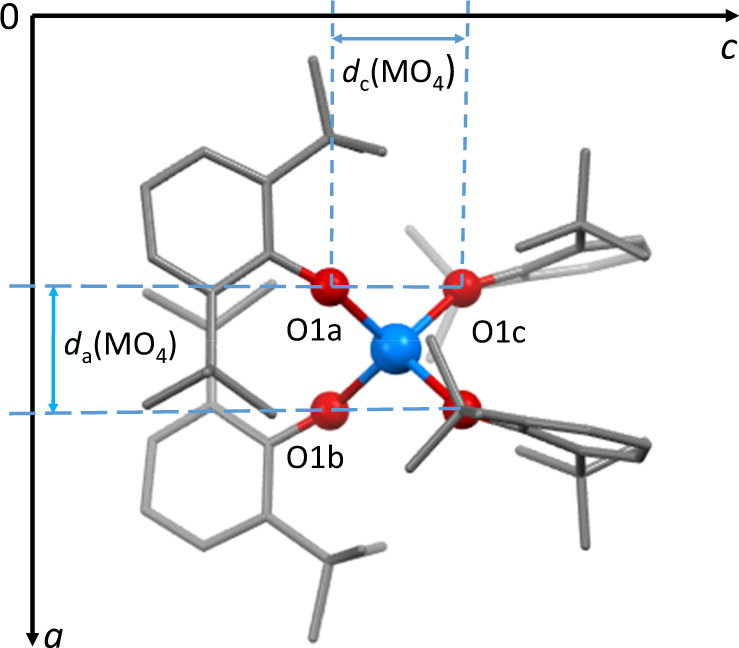
The structure of Np(OAr)_4_ in the solid state at ambient pressure. Hydrogen atoms and an identical molecule at the origin are omitted for clarity. The crystallographic axes and atoms used to monitor structural changes with pressure, and the projections (*d*) along the *a* and *c* axes that show the changes in the MO_4_ tetrahedron with pressure are indicated.

All three complexes (Fig. 2) crystallise in the tetragonal space group $I\bar{4}$ with crystallographic $\bar{4}$ (*S*_4_) symmetry and two molecules in the unit cell (*Z* = 2). Their central MO_4_ cores are close to tetrahedral, with shape indices (or continuous symmetry measures)^21,22^ relative to *T*_d_ symmetry of 0.065, 0.078 and 0.131 for M = Th, U and Np, respectively. The four symmetry-equivalent ^−^OAr ligands adopt a windmill conformation in projection, with M-O-C angles at oxygen in the range 156–157° (further structural data are available in Supplementary Table 5). The unit-cell volumes of Th(OAr)_4_ and U(OAr)_4_ are similar, but that of Np(OAr)_4_ is lower, reflecting the smaller radius of the Np atom (Supplementary Fig. 7). The effect of hydrostatic pressure on single crystals of all three compounds has been investigated, yielding data for structure determination to 4.30, 3.88 and 2.38 GPa for M = Th, U and Np, respectively.

**Fig. 3:**
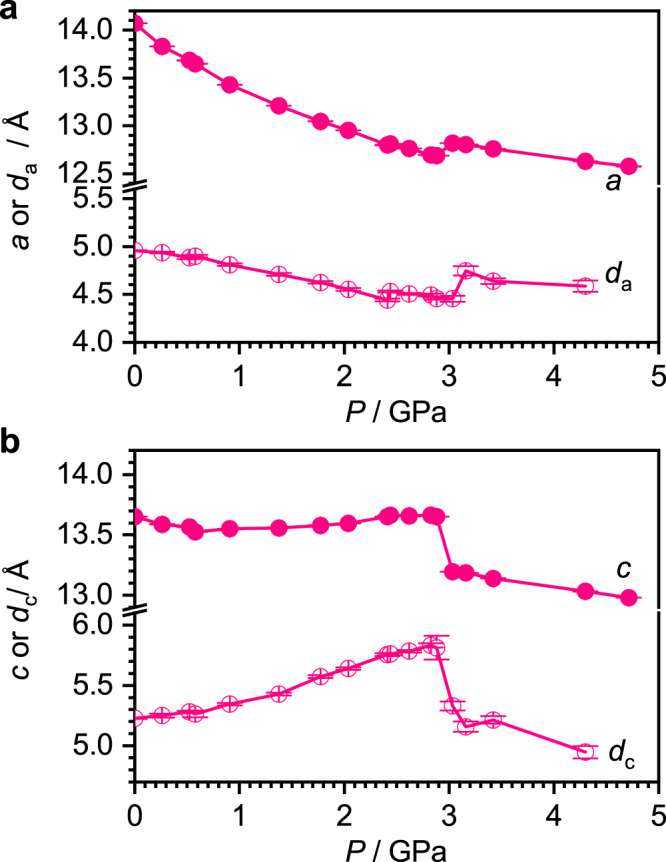
The effect of pressure on selected crystallographic metrics of Th(OAr)_4_. **a** Solid circles: the length of the unit cell parameter *a* (=*b*) as a function of pressure. Open circles: the projected contribution of the ThO_4_ moiety to the *a* axis length. **b** Corresponding plots for the *c* axis. Similar plots for U(OAr)_4_ and Np(OAr)_4_ are available in Supplementary Figs. 8 and 9. Calculation of the projected dimensions and their error bars is explained in the Supplementary Information, Section 1.17.

Four separate compression ranges can be identified for Th(OAr)_4_ and U(OAr)_4_ (Fig. 3 shows the trends for Th(OAr)_4_, with those for the other complexes shown in Supplementary Fig. 8): (i) between ambient pressure and 0.8 GPa *a* (=*b*) and *c* decrease. (ii) Between 0.8 GPa and 2.8 GPa the *c*-axis undergoes negative linear compressibility (i.e. it lengthens with pressure)^23^, while the *a* axis continues to shorten; there is no discontinuity in the unit cell volume, but the change from regimes (i) and (ii) can be interpreted as a second order phase transition. (iii) Between 2.8 and 3.2 GPa there is a first order phase transition signalled by a discontinuous drop in volume (Supplementary Fig. 7) with a pronounced decrease in *c* (referred to below as *c*-axis collapse), accompanied by a small increase in *a*. (iv) The compression becomes more isotropic above 3.2 GPa. The space group remains unchanged in all cases, and although the diffraction peaks broaden, they do not exhibit any splitting that might be associated with a reduction in crystallographic symmetry.

**Fig. 4:**
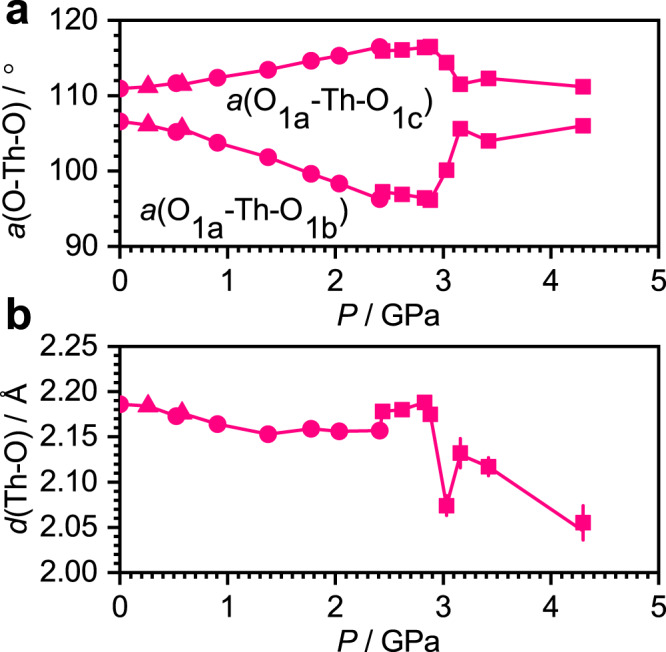
The influence of pressure on the structural parameters of Th(OAr)_4_. Data obtained with hydrostatic media Fluorinert FC70, Daphne 7373 and 1:1 pentane and isopentane are shown as triangles, circles and squares, respectively. **a** The two unique O-Th-O angles of the ThO_4_ unit (see Fig. 1 for labels). **b** The Th-O distance. Plots for the U and Np derivatives are available in Supplementary Fig. 10, which also includes data for the M-O-C angles and M-O-C-C torsion angles. Error bars taken from the crystallographic structure refinements are plotted, but are mostly smaller than symbols used for the data points.

The most prominent intramolecular effect of pressure between 0 and 3 GPa is on the O-M-O bond angles (M = Th or U). The angles O1a-M-O1b decrease with pressure (Fig. 4a) causing the projected height of the MO_4_ tetrahedron to increase in the *c* direction, leading to expansion between 0.8 and 2.8 GPa (open circles in Fig. 3b). Other intra- and intermolecular contributors to the length of the *c* axis decrease with pressure (Supplementary Fig. 9), and the negative linear compressibility in the Th and U systems is thus a reflection of the distortion of the MO_4_ tetrahedra.

**Fig. 5:**
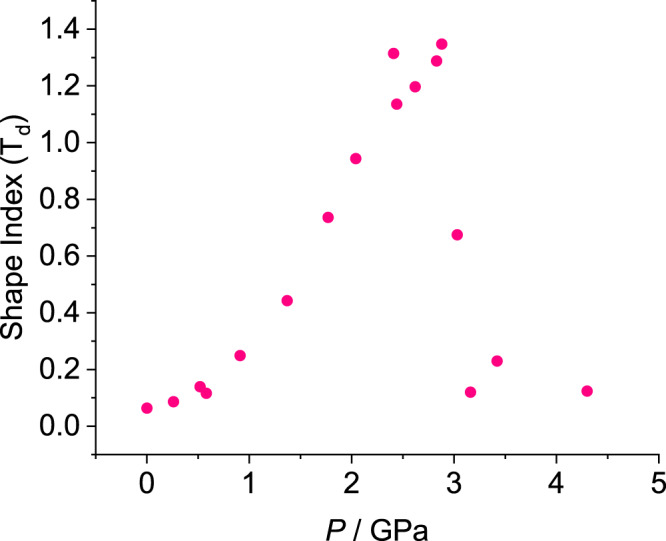
Shape index relative to ideal T_d_ point symmetry for Th(OAr)_4_ as a function of pressure. Plots for the U and Np complexes are available in Supplementary Fig. 11. A perfect tetrahedron would have a shape index relative to *T*_d_ point symmetry equal to zero.

The overall effect of the distortion is to cause the ThO_4_ and UO_4_ moieties to deviate steadily from ideal tetrahedral symmetry (Fig. 5), the shape index relative to *T*_d_ point group symmetry increasing from 0.064 to 1.348 for Th(OAr)_4_ between ambient pressure and 2.88 GPa; corresponding data for U(OAr)_4_ are 0.078 and 1.308 at 3.02 GPa.

Over the course of the phase transition at ~3 GPa, the trends exhibited by the Th and U complexes reverse, and there is an abrupt shortening of the mean M-O distance (Fig. 4b) accompanied by a degradation in the quality of the diffraction pattern (see Supplementary Information, Section 1.16). The Th-O and U-O bonds shorten from 2.175(3) Å to 2.074(10) Å (at 2.88 and 3.03 GPa) and 2.124(5) Å to 2.073(10) Å (at 2.88 and 3.02 GPa), respectively. The O1a-M-O1b angles increase, snapping-back to the values they had at ambient pressure (Fig. 4a) with the re-establishment of more ideal tetrahedral symmetry [shape indices: 0.675 for Th(OAr)_4_ at 3.03 GPa and 0.497 for U(OAr)_4_ at 3.32 GPa, the value lowering further with increasing pressure; Fig. 5]. The shortening of the M-O distance and increase in the O1a-M-O1b angle both occur between 2.88 and 3.03 GPa when M = Th, but appear to occur sequentially for M = U, at 2.88 GPa and 3.02 GPa for the U-O distance, but between 3.02 and 3.32 GPa for the bond angles and shape index.

The combination of the changes in the M-O distances and O1a-M-O1b angles lead to a sharp decrease in the projection of the MO_4_ tetrahedra in the *c*-direction. By contrast, the trends in the intermolecular contributions to the *c* axis length diminish more smoothly with pressure (Supplementary Fig. 9), so that the *c*-axis collapse is again a signature of intramolecular effects within the MO_4_ cores rather than intermolecular effects.

The increase in the projection along *c* of the MO_4_ tetrahedra in Th(OAr)_4_ and U(OAr)_4_ lead to a contraction of the dimensions along the *a* and *b* axes driven in part by the increase in the O1a-M-O1c angles (Figs. 3a and 4a). The complexes also rotate about the *c* axis with pressure driven by an interlocking of the tBu groups of neighbouring molecules which decreases the volume of interstitial intermolecular voids. A movie clip illustrating the rotation in Th(OAr)_4_ is available in Supplementary Movies 1 and 2 (viewed along the *b* and *c* directions, respectively); the development of the thermal ellipsoids with pressure, showing disordering of the ^t^Bu groups as they rotate, is available in Movie 3.

The complex Np(OAr)_4_, which is highly radioactive, was studied to a maximum pressure of 4.1 GPa. Data sets up to 2.38 GPa were suitable for structure analysis, but above this pressure only unit cell dimensions could be determined. Up to 2.38 GPa the O1a-Np-O1b and O1a-Np-O1c angles respectively decrease and increase, but the change in the shape index is less than half that seen for the Th and U complexes, changing from 0.031 at ambient pressure to 0.522 at 2.38 GPa. The height of the NpO_4_ tetrahedron projected along *c* increases, but is now over-ridden by the general contraction in the intermolecular interactions (Supplementary Fig. 9), so that negative linear compressibility of the *c*-axis is not observed. The Np-O bonds begin to shorten from 2.108(11) Å at 1.5 GPa to 2.075(15) Å at 2.03 GPa, reaching 2.054(8) Å at 2.38 GPa. The *c*-axis collapse is clearest above 2.38 GPa, but here too it is smaller in magnitude than for the Th and U complexes and occurs at lower pressure. Np(OAr)_4_ is thus seen to be more resistant to pressure than its Th and U analogues; it then exhibits the same first order phase transition, but at a lower pressure.

### The driving force of the phase transitions

**Fig. 6:**
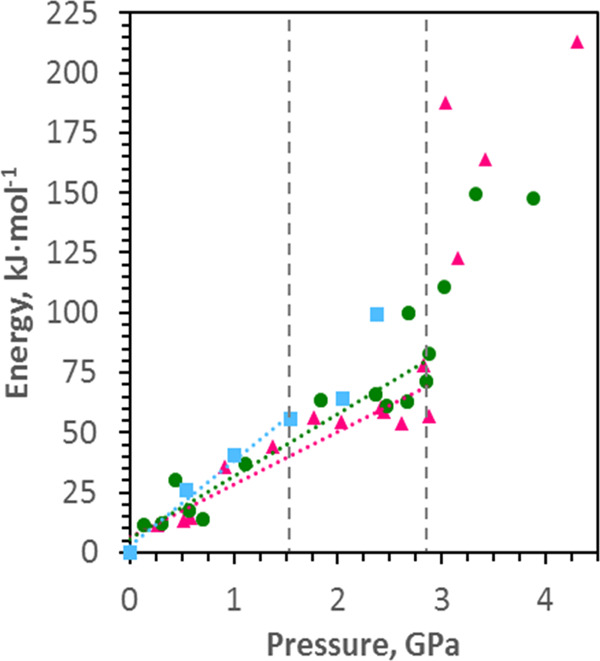
Energy changes for the M(OAr)_4_ complexes as a function of pressure for M = Th (pink triangles), U (green circles) and Np (blue squares). Linear regression analysis of values up to ca. 2.8 GPa for Th and U, and ca. 1.5 GPa for Np: *R*^2^ = 0.90, 0.88, and 0.98 for Th, U, and Np, respectively. The linear regression analysis extends in each case from ambient conditions up to the phase transition pressure (2.8 GPa for Th/U and 1.5 GPa for Np, indicated on the plot with vertical dashed lines.

To help interpret the structural changes caused by pressure, the M(OAr)_4_ systems were studied with scalar relativistic hybrid density functional theory using geometries in which the positions of the C, O and actinide atoms were taken from the crystal structures, and the H positions optimised. The energy changes for the M(OAr)_4_ complexes with pressure are shown in Fig. 6, with linear trend lines plotted within the low-pressure phases only.

The linearity of the trends below the transitions is an approximation, and there is a distinct flattening in the Th and U points between 2 and 3 GPa which parallels a similar flattening the O-M-O angles seen in Fig. 4a. The calculations are sensitive to statistical fluctuations in the crystal structures, and there is some scatter in all three datasets. Nevertheless, the energy changes with pressure follow the order Th < U < Np and above the phase transitions the internal energies increase markedly. The increase is offset by the *P*Δ*V* contribution to free energy resulting from a reduction in volume.

**Fig. 7:**
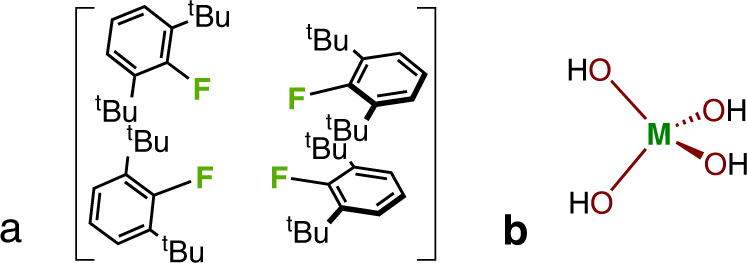
The two model systems used to explain the origin of the trends in M(OAr)_4_ geometry with pressure. **a** [FAr]_4_ used to understand the influence of inter-ligand repulsion. This system ensures a simple closed shell singlet electronic structure, isoelectronic with [(OAr)_4_]^4−^ and with similar electronegativity, avoiding the complicating effects associated with substantial negative electronic charge. **b** [M(OH)_4_] to understand the energy contribution from the MO_4_ core.

To determine the origin of these trends, the energies of two model systems were investigated. In the first, Fig. 7a, the influence of inter-ligand repulsion was estimated by calculating for each geometry the total energy of the four ligands with the [MO_4_] core replaced by four terminal fluorine atoms. The C and H positions were the same as in the energy calculations used in Fig. 6, but the F positions were allowed to optimise. In the second, Fig. 7b, the contribution from the distortion of the [MO_4_] core was estimated from the energy of M(OH)_4_ where the M and O positions were taken from the crystal structures and the aryl groups were replaced by geometry-optimised H atoms.

The [FAr]_4_ inter-ligand energies are strongly correlated with the total energies shown in Fig. 6, with correlation coefficients (*R*^2^) of 0.87, 0.86 and 0.94 for Th(OAr)_4_, U(OAr)_4_, and Np(OAr)_4_, respectively (Supplementary Fig. 12). By contrast, the energies in M(OH)_4_ are either weakly or even (for M = Np) negatively correlated (*R*^2^ = 0.30, 0.02, 0.75 for Th(OAr)_4_, U(OAr)_4_, and Np(OAr)_4_, respectively). This implies that the destabilisation of the complexes with pressure occurs as the four ligands are forced into closer proximity rather than because the geometry about the actinide is distorted. Interestingly, the dispersion energy contribution to the total energy change of [FAr]_4_ as a function of pressure becomes slightly more favourable (by up to c. 14 kJ/mol in the system based on the Np geometries), and hence the overall destabilisation results from increased Pauli repulsions as the ligands are brought together.

### Changes in the M-O bonds after the phase transitions

**Fig. 8:**
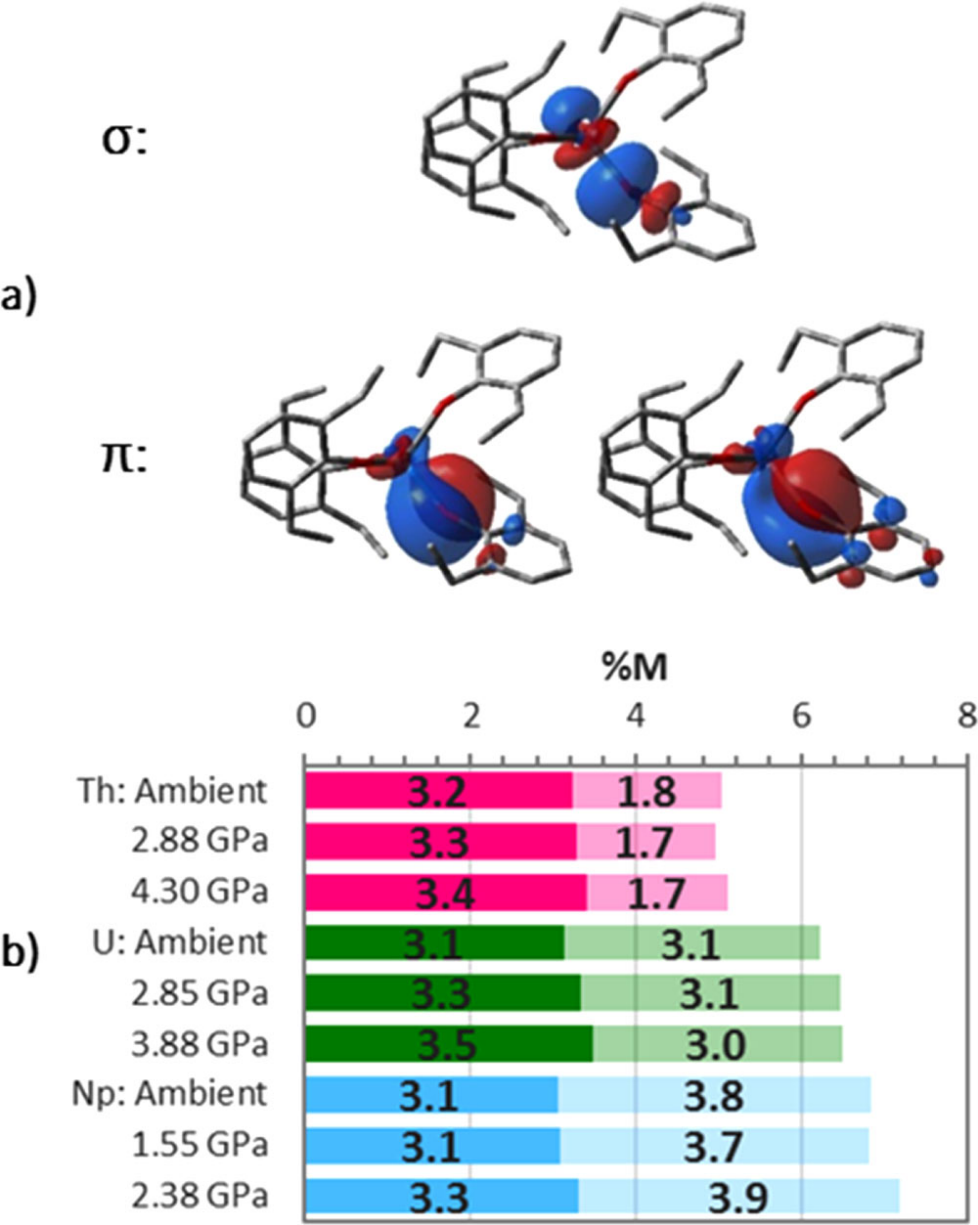
The nature of M-O bonding in M(OAr)_4_. **a** The σ- and π-type M-O bonding NLMOs for Np(OAr)_4_ at ambient pressure. The NLMOs for the other systems are similar. **b** The metal contribution (%M), averaged from σ and two π, are shown with relative contributions from d (dark), and f (light) orbitals depicted. There is no significant s- or p-orbital (<2 and <0.1% respectively) contribution. The raw data for this figure are available in Supplementary Table 10).

The nature of the M-O bonds before and after the shortening which occurs during the phase transition was probed with the QTAIM, and by analysis of the natural localised molecular orbitals (NLMOs) within the NBO framework. The latter finds the M-O bonds to be strongly O-polarised, suggesting mainly ionic character. The contributions from the actinides (%M) are 4–8% per bonding orbital in the order Th < U < Np. The M-O bonding interaction consists of one σ- and two π-type NLMOs (Fig. 8a), with the metal orbital contributions (Fig. 8b) showing that differences in the metal involvement in bonding is primarily the result of the periodic increase in f-orbital involvement (Th < U < Np). The differences in orbital character are most pronounced for the π-type orbitals (Supplementary Information, Table 8 and Fig. 13). The magnitude of the metal contribution to the NLMOs is associated with bond covalency and supports a covalency trend of Th < U < Np.

The results of the QTAIM calculations on the M(OAr)_4_ systems at different pressures are shown in Table 1. The trends in the electron density at the M-O bond critical points *ρ*_BCP_ and the delocalisation index *δ*(M, O) parallel the NLMO calculations in showing that at ambient pressure covalency follows the trend Th < U < Np^13^.

**Table 1:** Metal-oxygen distances *r*(M-O) (in Å) and QTAIM metrics (in atomic units (a.u.)) for M(OAr)_4_ (M = Th, U, Np) as a function of pressure

M	Pressure/GPa	*r*(M-O)/Å	Δ*r*/Å	*ρ*_BCP_	−(*G/V*)_BCP_	*δ*(M-O)
Th	Ambient	2.186 (2)		0.102	0.787	0.666
	2.88 GPa	2.175 (3)	−0.011	0.106	0.774	0.685
	4.30 GPa	2.055 (19)	−0.131	0.136	0.729	0.771
U	Ambient	2.145 (2)		0.108	0.797	0.734
	2.85 GPa	2.117 (5)	−0.028	0.116	0.781	0.768
	3.88 GPa	2.077 (13)	−0.067	0.127	0.762	0.802
Np	Ambient	2.119 (3)		0.114	0.791	0.769
	1.55 GPa	2.108 (11)	−0.011	0.120	0.783	0.789
	2.38 GPa	2.054 (8)	−0.065	0.137	0.763	0.842

*Δr* the change in the M-O distance relative to ambient pressure.

Data for all pressure are all systems shown in the Supplementary Information (Table 9 and Fig. 14).

The sudden shortening of the M-O distances at the phase transitions is accompanied by increases in %M, *ρ*_BCP_ and *δ*(M, O), as well as decreases in −(*G/V)*_BCP_ (Fig. 8 and Table 1). The change in −(*G/V)*_BCP_ indicates a decrease in the proportion of kinetic energy, a signature of covalent bonding resulting from increased electron sharing. The NLMO calculations indicate that enhancement of the metal contribution to the bonds occurs mainly in the σ orbitals via increased participation by the 6d, and the case of Np(OAr)_4_ the 5f, orbitals (Fig. 8 and Supplementary Fig. 13). The combination of these observations leads to the conclusion that the sudden shortening of the M-O bonds shifts their character towards overlap-driven covalency.

## Discussion

Compression of M(OAr)_4_ with M = Th, U initially leads to contraction of the O-M-O angles, distorting the molecules away from tetrahedral symmetry and elongating them along the *c* axis, but increasing inter-ligand repulsions. All three compounds undergo a phase transition, characterised by a collapse in the *c* unit cell dimension, between 2.8 and 3.3 GPa for M = Th, U, and above 2.3 GPa for M = Np (Fig. 3). At the phase transition the unit cell volume undergoes a discontinuous decrease, the M-O bonds contract sharply (Fig. 4b), the OMO angles readjust to nearer their ambient pressure values (Fig. 4a) and the near-tetrahedral symmetry of the MO_4_ core is re-established (Fig. 5).

The phase transitions are driven by a reduction in volume and relief of inter-ligand repulsions built up as the result of O-M-O angular changes. Prior to the phase transitions, the O-M-O angles in the Th and U change more than in the Np molecule. We suggest that the relative hardness of the Np system is because the ligands are already closer to each other at ambient pressure because Np is the smallest of the three actinides with shorter M-O bonds than in the Th and U compounds. Accordingly, the internal energy of the Np complex increases most rapidly as the pressure increases (Fig. 6).

The unmistakable discontinuities that occur in the M-O bond distances at the phase transitions are associated with a pressure-driven change in electronic structure where the primarily ionic, strongly polarised M-O bonds enter a more covalent regime through a greater interaction of the M 6d and 5f orbitals with the ligand O atoms. The availability of both 6d and 5f orbitals leads to minimal changes in metal-ligand orbital overlap as a function of structural distortion, and bonding changes occur at a low cost in energy: the metal 6d contributions increase for all, while the 5f contribution, which is already the larger component of the Np contribution, increases most for this metal.

## Methods

### Synthesis

A THF solution of KOAr (2.3 ml, 0.162 M) was added to 1 ml of a THF solution of NpCl_4_ (38.7 mg, 0.102 mmol), leading to an immediate colour change to deep red. The reaction mixture was allowed to stir for 16 h and volatiles removed under reduced pressure. Toluene (10 ml) was added and the red solution was isolated by syringe filtration (PTFE membrane, 0.45 µm) and concentrated under reduced pressure to ca. 1 ml to afford red crystals of the target product [Np(OAr)_4_] (94.4 mg, 0.0892 mmol) yield 87.8%. Further synthetic details are given in Section 1 of the Supplementary Information, along with comments on the targeted formation of analogous Zr and Ce derivatives.

### Crystallography

Single crystals of each material were loaded into Merrill-Bassett diamond anvil cells with a chip of ruby to enable the pressure to be measured from its fluorescence wavelength. Hydrostatic media were fluorinert FC70 (0–1 GPa), pentane-isopentane (1–5 GPa) and Daphne oil (0–2.5 GPa). For the Th and U derivatives data were collected on beamline I19 at Diamond Light Source using radiation of wavelength 0.4859 Å. For the Np derivative data were measured using Mo *K*α radiation on a Bruker Apex diffractometer. Data were collected to maximum pressures of 4.3, 3.9 and 4.1 GPa for the Th, U and Np systems, respectively. For the Np complex significant intensity was only available to atomic resolution up to 2.4 GPa. Above 2.4 GPa only unit cell dimensions could be determined: *a* = 12.6756(12) and *c* = 13.066(3) Å at 3.3 GPa and *a* = 12.5791(12) and *c* = 12.963(3) Å at 4.1 GPa. Crystal structures were solved using SHELXT^24^ and refined against *F*^2^ (CRYSTALS)^25^. Distances and angles within the ligands restrained in the all the refinements against high-pressure data to ambient pressure values; enhanced rigid body restraints were also applied to the anisotropic displacement parameters. The maximum crystallographic resolution achievable from the U and Th crystals decreased at the *c*-axis collapse, leading to increases in the uncertainty of the structural parameters at the highest pressures. Shape indices were calculate using the programme SHAPE^26^. Further details on the crystallographic analyses are available in the Supplementary Information. Crystallographic information files are available in the Cambridge Database with deposition numbers 1981193–1981195, 1981197–1981201, 1981204–1981216 and 2095929–095947.

### Computational procedures

The Gaussian 16 software package, revision A.03, was used for all density functional theory calculations^27^. The hybrid density functional approximation, PBE0, was used^28,29^, with dispersion treated by Grimme’s D3 correction and Becke-Johnson damping parameters (D3-BJ)^30–34^. Dunning’s basis sets of polarised triple-ζ quality were used for C, O and F atoms, and polarised double-ζ quality for H atoms^35,36^. The actinide atoms were treated with Stuttgart-Bonn small-core (60 electrons) relativistic effective core potentials, in combination with the associated segmented valence basis sets^37–39^. An ultrafine integration grid was used. Benchmarking of this methodology was described in ref. 13.

The NBO (7.0) software package was used to compute NLMOs to analyse actinide-oxygen bonding orbitals^40^. The CHOOSE option was employed to ensure M-O bonding was consistently defined by a σ and two π orbitals in all systems, to allow for direct comparison. The QTAIM bonding analysis was computed with the AIMAll software package^41^. The default settings were used to complete the electron density topology and integration of atomic basins. Occasionally, the more rigorous Promega(1) atomic integration algorithm was employed for atoms where numerical integration errors were present (defined by *L*(A) > 0.002 au).

## Supplementary information

Supplementary Information

Description of Additional Supplementary Files

Supplementary Movie 1

Supplementary Movie 2

Supplementary Movie 3

Supplementary Movie 4

Supplementary Movie 5

Supplementary Movie 6

Supplementary Movie 7

Supplementary Movie 8

Supplementary Movie 9

## Data Availability

The crystallographic datasets for the structures reported in this study have been deposited at the Cambridge Crystallographic Data Centre, under deposition numbers 1981193-1981216). Copies of the data can be obtained free of charge via https://www.ccdc.cam.ac.uk/structures/. All other data supporting the findings of this study and detailed experimental procedures and characterisation of compounds are available in the Supplementary Information files. Supplementary Movies 1–9 contain different views of the crystal structures of Th(OAr)_4_, U(OAr)_4_ and Np(OAr)_4_. Cartesian coordinates of all structures with optimised hydrogen positions and raw NMR spectroscopic data obtained during targeted syntheses of Zr(OAr)_4_ and Ce(OAr)_4_ can be found at dx.doi.org/10.17632/2mhj4xy8d4.1.

## References

[1] Casati N, Kleppe A, Jephcoat AP, Macchi P (2016). Putting pressure on aromaticity along with in situ experimental electron density of a molecular crystal. Nat. Comm..

[2] Prescimone A (2008). [Mn6] under pressure: a combined crystallographic and magnetic study. Angew. Chem. Int. Ed..

[3] Price AN (2022). Contrasting behaviour under pressure reveals the reasons for pyramidalization in tris(amido)uranium(III) and tris(arylthiolate) uranium(III) molecules. Nat. Comm..

[4] Arnold PL (2015). Characterizing pressure-induced uranium C-H agostic bonds. Angew. Chem. Int Ed.

[5] Sperling JM (2020). Compression of curium pyrrolidine-dithiocarbamate enhances covalency. Nature.

[6] Cantat T (2008). Evidence for the involvement of 5f orbitals in the bonding and reactivity of organometallic actinide compounds: Thorium(IV) and Uranium(IV) Bis(hydrazonato) complexes. J. Am. Chem. Soc..

[7] Yang P, Warnke I, Martin RL, Hay PJ (2008). Theoretical studies of the sp^2^ versus sp^3^ C−H bond activation chemistry of 2-Picoline by (C_5_Me_5_)_2_An(CH_3_)_2_ complexes (An = Th, U). Organometallics.

[8] Su J (2018). Energy-degeneracy-driven covalency in actinide bonding. J. Am. Chem. Soc..

[9] Formanuik A (2017). Actinide covalency measured by pulsed electron paramagnetic resonance spectroscopy. Nat. Chem..

[10] Smiles DE, Wu G, Hrobárik P, Hayton TW (2016). Use of ^77^Se and ^125^Te NMR spectroscopy to probe covalency of the actinide-chalcogen bonding in [Th(E_*n*_){N(SiMe_3_)_2_}_3_]^−^ (E = Se, Te; *n* = 1, 2) and their oxo-Uranium(VI) congeners. J. Am. Chem. Soc..

[11] Kaltsoyannis N (2013). Does covalency increase or decrease across the actinide series? Implications for minor actinide partitioning. Inorg. Chem..

[12] Kerridge A (2017). Quantification of f-element covalency through analysis of the electron density: insights from simulation. Chem. Comm..

[13] Berryman VEJ (2019). Computational analysis of M–O covalency in M(OC_6_H_5_)_4_ (M = Ti, Zr, Hf, Ce, Th, U). Dalton Trans..

[14] Kaltsoyannis N (2018). Transuranic computational chemistry. Chem. Eur. J..

[15] Bell NL, Maron L, Arnold PL (2015). Thorium mono- and bis(imido) complexes made by reprotonation of *cyclo*-metalated amides. J. Am. Chem. Soc..

[16] Cowie BE, Purkis JM, Austin J, Love JB, Arnold PL (2019). Thermal and photochemical reduction and functionalization chemistry of the uranyl dication, [U^VI^O_2_]^2+^. Chem. Rev..

[17] Hayton TW (2005). Synthesis of imido analogs of the uranyl ion. Science.

[18] Van Der Sluys WG, Sattelberger AP, Streib WE, Huffman JC (1989). Tetrakis(2,6-di-t-butylphenoxy)uranium(IV): the first structurally characterized neutral homoleptic aryloxide complex of uranium(IV). Polyhedron.

[19] Berg JM (1992). Early actinide alkoxide chemistry. Synthesis, characterization, and molecular structures of thorium(IV) and uranium(IV) aryloxide complexes. J. Am. Chem. Soc..

[20] Cantat T, Scott BL, Kiplinger JL (2010). Convenient access to the anhydrous thorium tetrachloride complexes ThCl_4_(DME)_2_, ThCl_4_(1,4-dioxane)_2_ and ThCl_4_(THF)_3.5_ using commercially available and inexpensive starting materials. Chem. Comm..

[21] Pinsky M, Avnir D (1998). Continuous symmetry measures. 5. The classical polyhedra. Inorg. Chem..

[22] Alvarez S (2005). Shape maps and polyhedral interconversion paths in transition metal chemistry. Coord. Chem. Rev..

[23] Cairns AB, Goodwin AL (2015). Negative linear compressibility. Phys. Chem. Chem. Phys..

[24] Sheldrick G (2015). SHELXT—integrated space-group and crystal-structure determination. Acta Cryst..

[25] Betteridge PW, Carruthers JR, Cooper RI, Prout K, Watkin DJ (2003). CRYSTALS version 12: software for guided crystal structure analysis. J. Appl Cryst..

[26] Llunell, M., Casanova, D., Cirera, J., Alemany, P. & Alvarez, S. SHAPE. Version 2.1. Universitat de Barcelona, Spain (2013).

[27] Frisch, M. J. et al. Gaussian 16, Revision A.03, Gaussian, Inc., Wallingford CT, USA (2016).

[28] Ernzerhof M, Scuseria GE (1999). Assessment of the Perdew–Burke–Ernzerhof exchange-correlation functional. J. Chem. Phys..

[29] Adamo C, Barone V (1999). Toward reliable density functional methods without adjustable parameters: the PBE0 model. J. Chem. Phys..

[30] Grimme S, Antony J, Ehrlich S, Krieg H (2010). A consistent and accurate ab initio parametrization of density functional dispersion correction (DFT-D) for the 94 elements H-Pu. J. Chem. Phys..

[31] Johnson ER, Becke AD (2005). A post-Hartree–Fock model of intermolecular interactions. J. Chem. Phys..

[32] Johnson ER, Becke AD (2006). A post-Hartree-Fock model of intermolecular interactions: Inclusion of higher-order corrections. J. Chem. Phys..

[33] Grimme S, Ehrlich S, Goerigk L (2011). Effect of the damping function in dispersion corrected density functional theory. J. Comp. Chem..

[34] Becke AD, Johnson ER (2005). A density-functional model of the dispersion interaction. J. Chem. Phys..

[35] Dunning TH (1989). Gaussian basis sets for use in correlated molecular calculations. I. The atoms boron through neon and hydrogen. J. Chem. Phys..

[36] Kendall RA, Dunning TH, Harrison RJ (1992). Electron affinities of the first‐row atoms revisited. Systematic basis sets and wave functions. J. Chem. Phys..

[37] Küchle W, Dolg M, Stoll H, Preuss H (1994). Energy‐adjusted pseudopotentials for the actinides. Parameter sets and test calculations for thorium and thorium monoxide. J. Chem. Phys..

[38] Cao X, Dolg M, Stoll H (2003). Valence basis sets for relativistic energy-consistent small-core actinide pseudopotentials. J. Chem. Phys..

[39] Cao X, Dolg M (2004). Segmented contraction scheme for small-core actinide pseudopotential basis sets. J. Mol. Struct. THEOCHEM.

[40] Glendening ED, Landis CR, Weinhold F (2019). NBO 7.0: New vistas in localized and delocalized chemical bonding theory. J. Comp. Chem..

[41] Keith, T. A. AIMALL. Version 19.02.13. Gristmill Software, Overland Park KS, USA (2019).

