# Supplementary material for: Covalent bond shortening and distortion induced by pressurization of thorium, uranium, and neptunium tetrakis aryloxides
Source: Nat Commun. 2022 Oct 7;13:5923. doi: 10.1038/s41467-022-33459-7 (PMC9546877; doi:10.1038/s41467-022-33459-7)
Supplement: Supplementary file 1 — Supplementary Information [file 41467_2022_33459_MOESM1_ESM.pdf]

Supplementary Information

High pressure and the control of covalency in actinide complexes $M(OAr)_4$ (M = Th, U, Np)

1: Supplementary Methods (Pages 2-12)

1.1 General Synthetic Procedures
1.2 General notes
1.3 Synthesis of KOAr
1.4 Synthesis of $Ce(N^iPr_2)_4$
1.5 Synthesis of $[U(N(SiMe_3)SiMe_2CH_2)(N")_2]$ (N" = $N(SiMe_3)_2$)
1.6 Synthesis of $ThCl_4(DME)_2$
1.7 Synthesis of $ZrBn_4$ (Bn = CH_2Ph)
1.8 Synthesis of $NpCl_4$
1.9 Synthesis of $U(OAr)_4$
1.10 Synthesis of $Th(OAr)_4$
1.11 Synthesis of $Np(OAr)_4$
1.12 Reaction to target $Zr(OAr)_4$
1.13 Reaction to target $Ce(OAr)_4$
1.14 Percent Buried Volume Calculations using SAMBVCA 2.0
1.15 Comparison of the estimated %*V*_Bur above with those calculated for $U(OAr)_4$ for CDS structure 1185610.
1.16 Crystal structure determinations
1.17 Projection analysis of crystallographic data
1.18 Shape index calculations

2: Supplementary Tables (Pages 13-27)

3: Supplementary Figures (Pages 28-34)

4: Supplementary References (Pages 34-36)

1. Supplementary Methods

1.1 General Synthetic Procedures

All manipulations were carried out under a dry, oxygen-free atmosphere of dinitrogen using standard Schlenk and glovebox techniques. THF, toluene and hexane were degassed and purified by passage through activated alumina towers and stored over 4 Å molecular sieves. Deuterated benzene was boiled over potassium, freeze-pump-thaw degassed three times and vacuum transferred prior to use. All gases were supplied by BOC gases UK. All glassware items, cannulae and Fisherbrand 1.2 µm retention glass microfibre filters were dried in a 150 °C oven overnight before use. The phenol HOAr (OAr = 2,6-di-tert-butylphenoxide) was sublimed prior to use.

Caution! Compounds containing the ^{237}Np isotope represent a potential health risk owing to α emission ($Q\alpha$ = 4.958 MeV, $t_{1/2}$ = 2.14 × 10^6 years). ^{237}Np decays to ^{233}Pa ($t_{1/2}$ = 26.97 days, a = 21 kCi g^{-1}), a β emitter (Q- = 0.570 MeV) which can result in radiation exposure due to penetration of the gloves and skin. Handling Np isotopes must only be undertaken in a properly regulated and controlled radiological facility.

1.2 General notes

The manipulations with Np radionuclides were conducted in the radiochemical laboratories at the Joint Research Centre (JRC) – Karlsruhe, Germany. Unsealed transuranium compounds were manipulated in dinitrogen filled (99+%), negative-pressure radiological gloveboxes. The glovebox for preparative chemistry was fitted with an automated dual vacuum/argon manifold and standard Schlenk techniques were used.

1.3 Synthesis of KOAr[1]

Potassium hexamethyldisilazide (1.81g, 9.1mmol) was added to a stirring colorless solution of 2,6-ditertbutylphenol (2.00g, 9.5mmol) in thf (70mL) at room temperature. The reaction mixture was stirred overnight; the volatiles were subsequently removed *in vacuo*. The pale pink residue was washed with pentane (30 mL), collected on a glass sintered frit and dried under reduced pressure to give KOAr as a solid in essentially quantitative yield (2.15 g, 8.8 mmol). The ^{1}H-NMR spectrum in thf-d^8 agrees with the literature.[1]

1.4 Synthesis of $Ce(N^iPr_2)_4$[2]

$CeCl_3$ (100 mg, 0.41 mmol) was stirred in hexane (20 mL) with 1 mL of thf. After 1 hour, lithium diisopropylamide (175 mg, 1.61 mmol) was added and stirred for 36 hours. The solvent was removed *in vacuo* and the remaining solid was extracted into toluene (10 mL). Hexachloroethane (47 mg, 0.20 mmol) was added with stirring; gas evolution was observed and the solution turned dark blue. After stirring for 2 hours the reaction mixture was filtered to remove LiCl and the solution was concentrated *in vacuo*. A few drops of hexane were added to induce precipitation of $Ce(N^iPr_2)_4$ as a blue powder in 88 % yield (196 mg, 0.36 mmol). The ^{1}H-NMR spectrum in C_6D_6 agrees with the literature.[2]

1.5 Synthesis of $[U(N(SiMe_3)SiMe_2CH_2)(N'')_2]$ (N" = $N(SiMe_3)_2$) [3]

$UI_4(Et_2O)_2$ (5.0 g, 5.7 mmol) and sodium hexamethyldisilazide (4.1 g, 22 mmol) were stirred together at room temperature in toluene (100 mL) for 30 minutes prior to reflux at 110°C overnight. The volatiles were removed *in vacuo* and the remaining solids were extracted in hexane (50 mL), followed by filtration. The solution was concentrated to approximately 10 mL in volume; the product was crystallised from this solution as orange crystals at -35°C overnight in 26 % yield (1.05g, 1.5 mmol). The ^{1}H-NMR spectrum in C_6D_6 agrees with the literature.[3]

1.6 Synthesis of $ThCl_4(DME)_2$ [4]

Thorium nitrate hexahydrate (20 g, 34 mmol) was boiled in 12M HCl (100 mL) with nitrogen blowing over the reaction mixture until an oily yellow residue remained after approximately 16 hours. This material was dried under vacuum for 36 hours to give an off-white powder. This residue was suspended in DME (120 mL) with stirring, followed by the dropwise addition of trimethylsilyl chloride (80 mL). The mixture was then refluxed

with stirring at 60°C for 4 days. The volatiles were removed *in vacuo* to give a sticky white residue; this was then dried under vacuum at 120°C overnight to give the product as a free-flowing white powder (15.1 g, 27 mmol, 80% yield).

1.7 $ZrBn_4$ (Bn = CH_2Ph) [5]

To a cooled stirred mixture of $ZrCl_4$ (1.15 g, 5.0 mmol) in toluene (25 mL) was added benzyl magnesium chloride solution (25 mL, 1.0M in Et_2O, 25mmol) at -78°C. The reaction mixture was covered in foil to protect against degradation by light and allowed to warm to room temperature overnight, after which it was stirred at room temperature for a further 24 hours. The volatiles were removed *in vacuo* and the remaining brown solid was extracted into toluene (25 mL) and concentrated to incipient crystallization (approx. 5 mL in volume). The liquors were stored at -20°C and yielded orange crystals of $ZrBn_4$ after one week in 35% yield (0.8 g, 1.8 mmol). The ^{1}H-NMR spectrum in C_6D_6 agrees with the literature.[5]

1.8 $NpCl_4$

$^{237}NpCl_4$ was prepared by chlorination of $^{237}NpO_2$ with a $Cl_2/CCl_4/Ar$ gas mixture according to a modified literature method.[6, 7]

1.9 Synthesis of $U(OAr)_4$

A 200 mL ampoule was charged with a stir bar, $(N'')_2U[\kappa^2\text{-}(N,C)\text{-}N(SiMe_3)SiMe_2CH_2]$ (0.589 g, 0.820 mmol), HOAr (0.675 g, 3.28 mmol), and toluene (20 mL). The solution was heated at 110 °C for 12 h, after which the solvent was removed in vacuo to give $U(OAr)_4$ as a dark yellow solid (0.611 g, 0.577 mmol, 70%). ^{1}H NMR spectrum collected in C_6D_6 were consistent with the data previously reported for complex $U(OAr)_4$.[8] Single crystals suitable for X-ray diffraction could be obtained by crystallization from a saturated toluene solution at -35 °C.

1.10 Synthesis of $Th(OAr)_4$

A 250-mL sidearm flask equipped with a stir bar was charged with $ThCl_4(DME)_2$ (0.83 g, 1.50 mmol) and THF (15 mL). A THF (10 mL) solution of KOAr (1.47 g, 6.02 mmol) was added dropwise at room temperature with stirring and the reaction mixture stirred for 12 h. After isolation via cannula filtration and drying in vacuo, $Th(OAr)_4$ was isolated as a colourless solid (1.35 g, 1.28 mmol, 85%).The ^{1}H NMR spectrum collected in C_6D_6 is consistent with the data previously reported for $Th(OAr)_4$.[8] Single crystals suitable for X-ray diffraction could be obtained by crystallization from the slow diffusion of hexane into a THF solution of $Th(OAr)_4$.

1.11 Synthesis of $Np(OAr)_4$

A THF solution of KOAr (2.3 ml, 0.162 M) was added to 1 ml THF solution of $NpCl_4$ (38.7 mg, 0.102 mmol), leading to an immediate colour change of the reaction mixture to deep red. The reaction mixture was allowed to stir for further overnight and the solvent stripped under reduced pressure. Then 10 ml toluene was added and the red solution was isolated by syringe filtration (PTFE membrane, 0.45 µm) and concentrated under reduce pressure to ca. 1 mL to afford red crystals of the target product $Np(OAr)_4$ (94.4 mg, 0.0892 mmol, 87.8%).

1.12 Reaction to target $Zr(OAr)_4$

To a yellow solution of $ZrBn_4$ (25 mg, 0.055 mmol) in C_6D_6 (0.4 mL) was added HOAr (45 mg, 4 eq., 0.22 mmol). The mixture was shaken until all HOAr had dissolved (approximately one minute). The reaction was monitored by ^{1}H-NMR (500.1 MHz, C_6D_6, 300 K); after 3 hours at room temperature one equivalent of HOAr had been consumed, and a species with resonances assigned to the product $Zr(OAr)Bn_3$ was observed in NMR spectra, along with one equivalent of toluene (Supplementary Figures 1 and 2). After three days resonances that could be attributed to the target $Zr(OAr)_4$ were still not visible in the spectra, and since the Zr-Bn groups are unstable with respect to the formation of Bn radicals under more forcing reaction conditions, further attempts to prepare $Zr(OAr)_4$ by this method were not pursued. Our %*V*_Bur calculations (see below) agree, suggesting

that it is likely too sterically congested around the Zr^{4+} cation to be isolable. Raw ^{1}H and ^{13}C-NMR data are available at dx.doi.org/10.17632/2mhj4xy8d4.1.

^{1}H-NMR (500 MHz, 300 K, C_6D_6): δ 7.31 (d, *J* = 7.9 Hz, 2H, Zr(OAr)Bn_3, meta OAr), 7.17 (d, *J* = 7.9 Hz, 6H, HOAr, meta), 7.12 (t, *J* = 7.5 Hz, 2H, toluene), 7.08 (t, *J* = 7.7 Hz, 6H, Zr(OAr)Bn_3, Bn meta), 7.05 (d, *J* = 7.6 Hz, 1H, toluene), 7.01 (d, *J* = 7.6 Hz, 2H, toluene), 6.97 – 6.91 (m, 4H, overlapped resonances for Zr(OAr)Bn_3, OAr para and Bn para), 6.85 (t, *J* = 7.8 Hz, 3H, HOAr, para), 6.66 (d, *J* = 7.7 Hz, 6H, Zr(OAr)Bn_3, Bn ortho), 4.92 (s, 3H, HOAr, -OH), 2.17 (s, 6H, Zr(OAr)Bn_3, methylene), 2.11 (s, 3H, toluene, methyl), 1.45 (s, 18H, Zr(OAr)Bn_3, OAr ^{t}Bu), 1.35 (s, 54H, HOAr, ^{t}Bu).

^{13}C-NMR (126 MHz, C_6D_6): δ 163.1 (q, Zr(OAr)Bn_3), 154.3 (q, HOAr), 141.1 (q, Zr(OAr)Bn_3), 138.8 (q, Zr(OAr)Bn_3), 137.9(q, toluene), 136.1(q, HOAr), 130.4 (CH, Zr(OAr)Bn_3), 129.3 (toluene), 128.9 (CH, Zr(OAr)Bn_3), 128.6 (toluene), 125.7 (CH, Zr(OAr)Bn_3), 125.4 (CH, HOAr), 124.4 (CH, Zr(OAr)Bn_3), 121.9 (CH, Zr(OAr)Bn_3), 120.4 (CH, HOAr), 73.9 (CH_2, Zr(OAr)Bn_3), 35.3(q, Zr(OAr)Bn_3), 34.4 (q, HOAr), 31.5 (CH_3, Zr(OAr)Bn_3), 30.5 (CH_3, HOAr), 21.5 (CH_3, toluene).

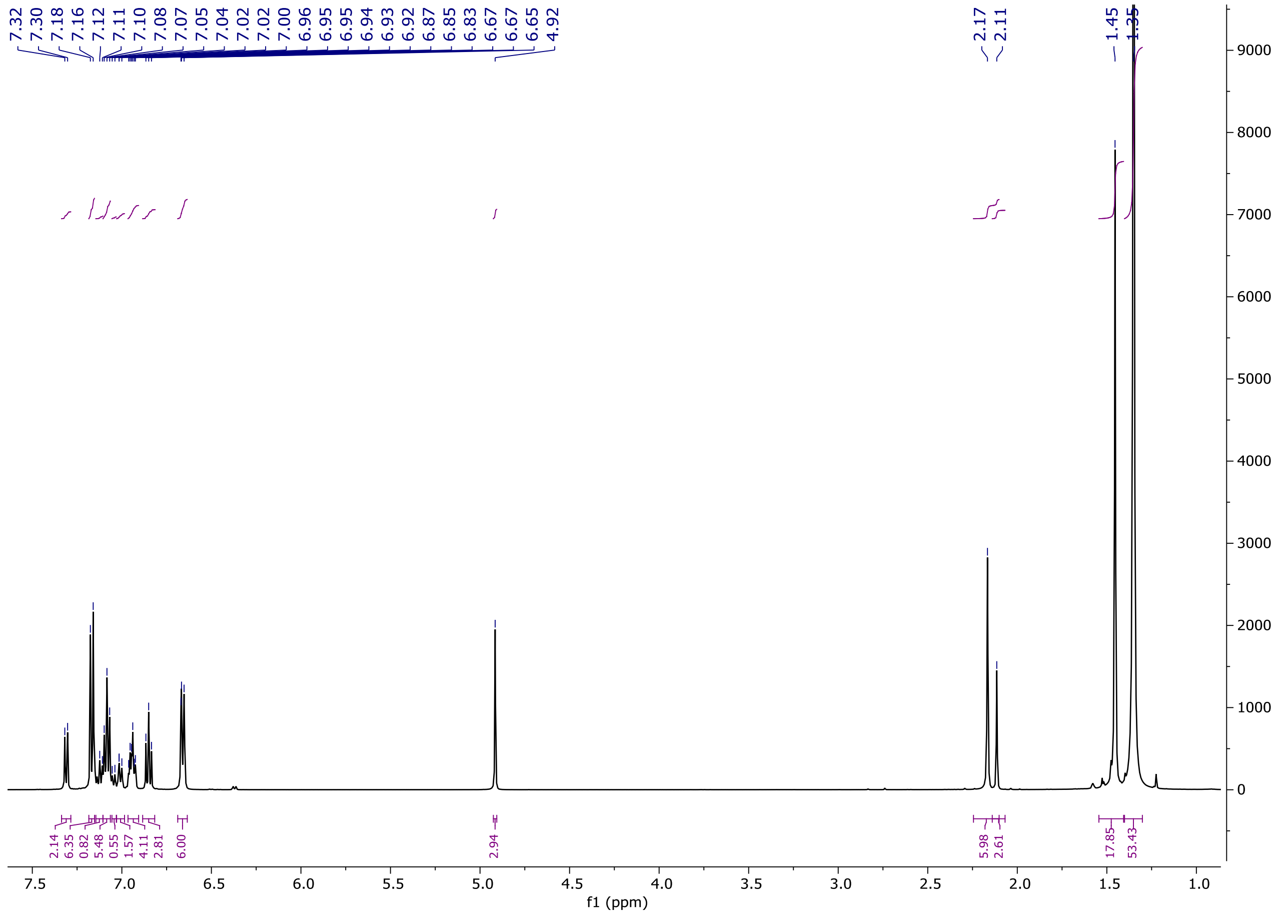

Supplementary Figure 1. ^{1}H-NMR (500 MHz, 300K, C_6D_6) of the reaction mixture targeting $Zr(OAr)_4$ after 3 h.

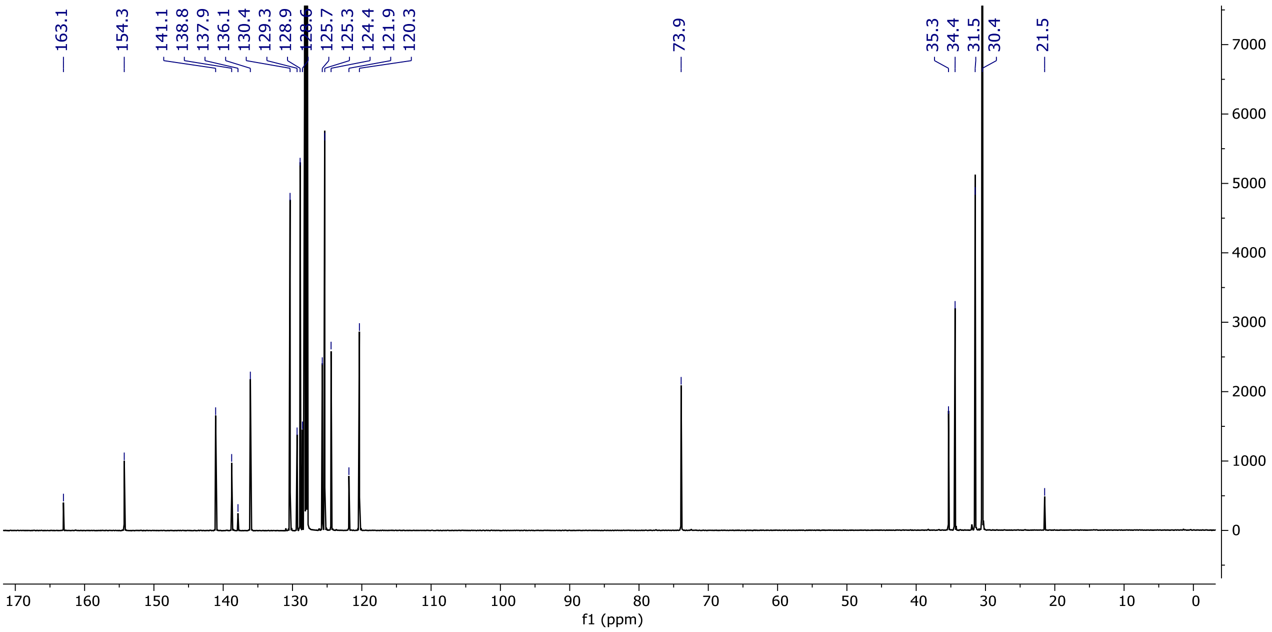

Supplementary Figure 2. ^{13}C-NMR (126 MHz, 300K, C_6D_6) of the reaction mixture of $ZrBn_4$ and HOAr after 3 hours.

1.13 Reaction to target $Ce(OAr)_4$

To $Ce(N^iPr_2)_4$ (8 mg, 0.015 mmol) in C_6D_6 (0.2 mL) was added a solution of HOAr (12.2 mg, 0.059 mmol) in C_6D_6 (0.2 mL) at room temperature. A colour change from purple to brown was observed over 16 hours. The reaction was followed by ^1H-NMR (500.1 MHz, C_6D_6, 300 K) over 6 days; resonances corresponding to those reported for $Ce(OAr)_3$ were observed to appear and grow in over this period,[9] along with resonances presumed to correspond to other Ce(III) containing species (see Supplementary Figures 3 and 4), as these also appear in the paramagnetic region of the spectrum, and resonances corresponding to HN^iPr_2.[10] The apparent reduction of Ce(IV) to Ce(III) is presumed to occur via Ce-X bond homolysis (X= O or N). it is therefore not possible to prepare $Ce(OAr)_4$ directly by this method, and given the presence of elongated U-O bonds in the U^{IV} analogue, we did not pursue routes including an additional oxidant.[11] Raw ^1H-NMR data are available at dx.doi.org/10.17632/2mhj4xy8d4.1.

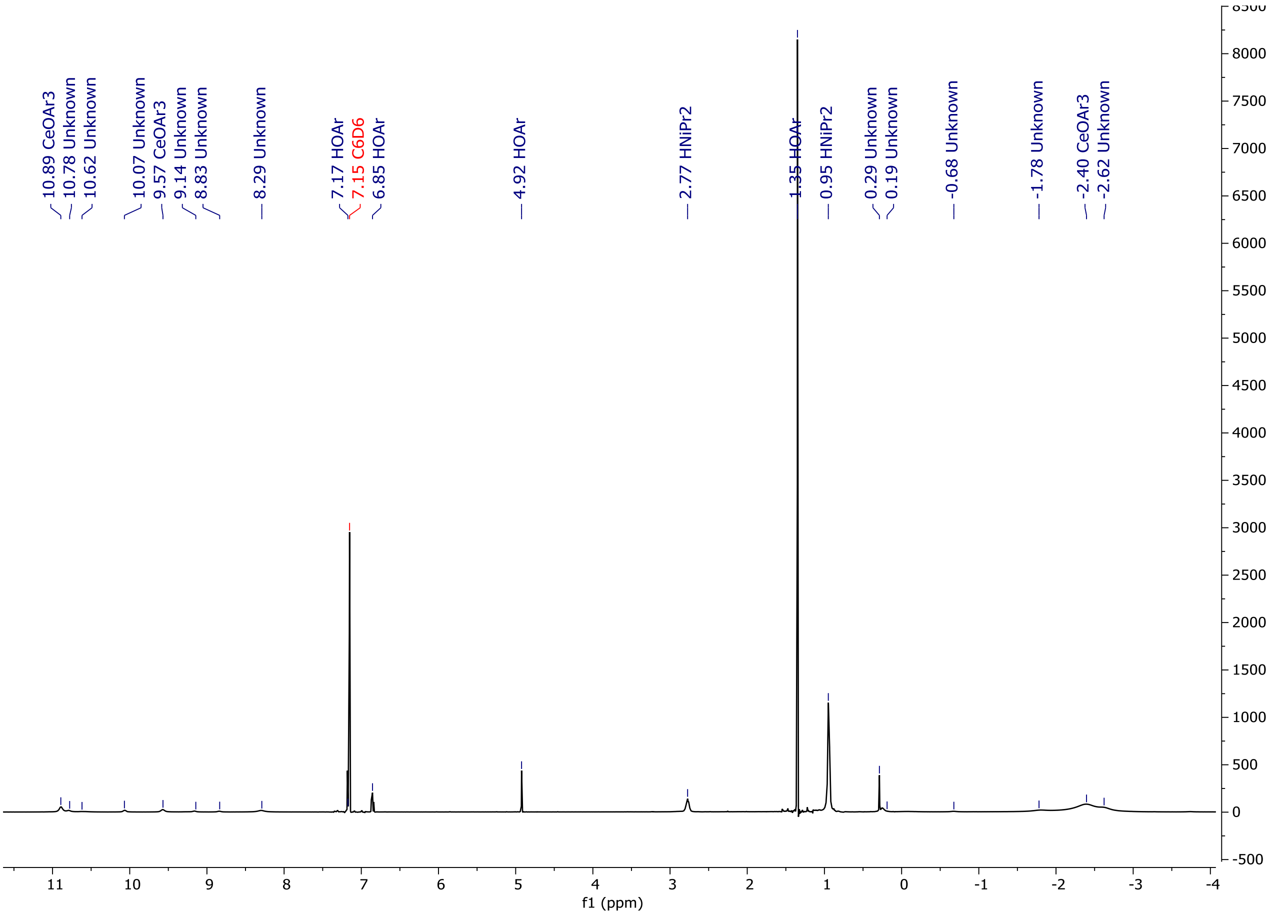

Supplementary Figure 3. ^{1}H-NMR (500 MHz, 300K, C_6D_6) of the reaction mixture targeting $Ce(OAr)_4$ after 6 days

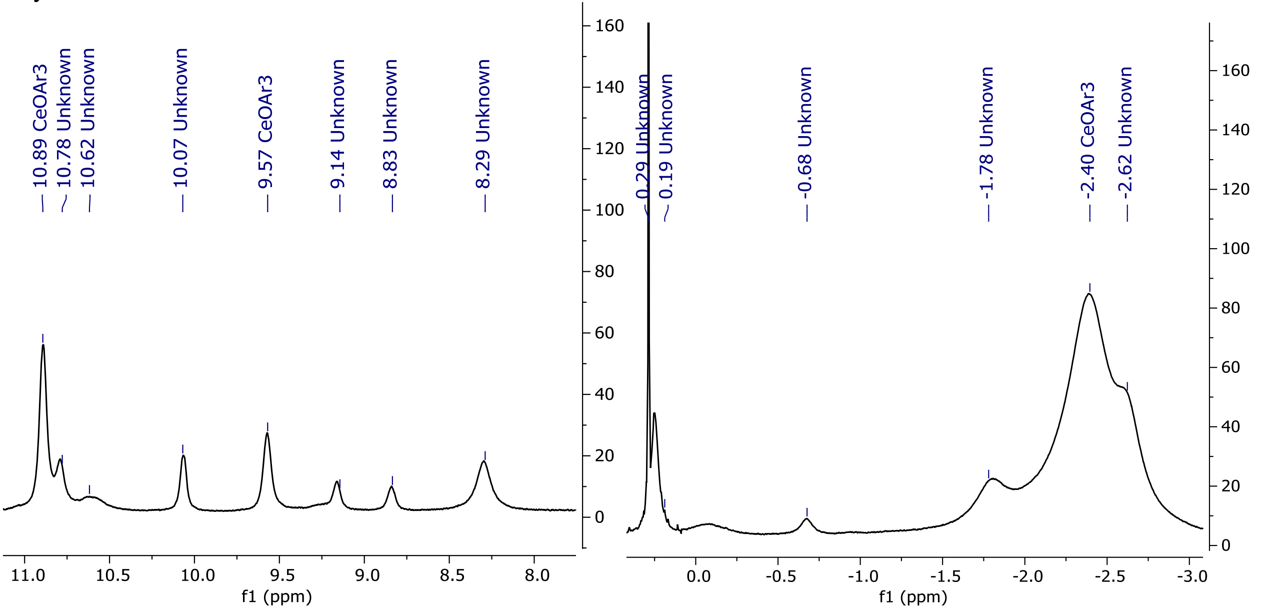

Supplementary Figure 4. Sections of the ^{1}H-NMR (500 MHz, 300K, C_6D_6) spectrum of the reaction mixture targeting $Ce(OAr)_4$ after 6 days

1.14 Percent Buried Volume Calculations using SAMBVCA 2.0[12]

Method of estimating the percent of buried volume about the metal centre for hypothetical $Zr(OAr)_4$ and $Ce(OAr)_4$ complexes.

The SAMBVCA applet 2.0 was used to estimate the volume each OAr ligand occupies around the metal centre within a sphere of 3.5 Å radius, centred on the metal centre (%*V*_Bur).[12] All default parameters in the SAMBVCA applet were used as found, unless otherwise stated. The coordinates used for the OAr ligand were taken from CCDC structure 1185610 and are given below in Supplementary Table 1.[13] Approximations used include the imposition of a 180° M-O-C bond angle (which reduces the volume required by the OAr ligand) for all metal complexes and using ligand coordinates for a single OAr ligand from $U(OAr)_4$ CSD structure 1185610, without optimising its geometry for the other metal centres. Approximations are discussed below.

Supplementary Table 1: Coordinates used for the OAr ligand

Atom	#	*x*	*y*	*z*
O	0	5.84442	5.77972	7.95796
C	1	5.55185	5.15378	9.10936
C	2	5.05954	5.92179	10.25272
C	3	4.89637	5.23396	11.46712
C	4	5.10314	3.88081	11.5958
C	5	5.41541	3.16063	10.45512
C	6	5.59827	3.74578	9.22329
C	7	5.82192	2.86806	7.98476
C	8	4.69945	3.14797	6.95936
C	9	7.22992	3.07623	7.37756
C	10	5.74174	1.36159	8.33193
C	11	4.6671	7.42403	10.30097
C	12	5.34086	8.13437	11.49125
C	13	3.12968	7.47327	10.43099
C	14	5.09049	8.32707	9.11606

To obtain the %*V*_Bur of a single OAr ligand the following steps were followed for each metal(IV) ion;

1) The OAr.xyz file was loaded into the applet.
2) O(0) was set as the centre of the sphere (i.e. the metal atom).
3) The z-axis was defined by clicking on C(1). (This will artificially set the M-O-C angle to 180°).
4) The xz-plane was defined by clicking on C(2) and C(6).
5) The Bondi van der Waals radii were scaled by 1.17 (applet default).
6) The sphere radius was set to 3.5 Å (applet default).
7) The distance of the coordination point from the centre of the sphere was set to the average for tetra-coordinated M-OAr complexes. (This was determined from CSD database v5.40 (+3 updates) and Mogul geometry search of these databases; Zr-OAr 1.913 Å, Ce-OAr 2.119 Å, Th-OAr 2.192 Å, U-OAr 2.119 Å).
8) The mesh spacing for numerical integration was set to 0.10 (applet default) and the job was then submitted to the applet.
9) The %*V*_Bur for $MOAr_4$ was then estimated by multiplying the calculated %*V*_Bur for one OAr by four. This was deemed a reasonable approximation as the observed structures for $U(OAr)_4$ and $Th(OAr)_4$ have I-4 symmetry at ambient pressure.

The %*V*_Bur estimates for MOAr and $M(OAr)_4$ (M = Zr, Ce, Th and U) are given in Supplementary Table 2 below.

Supplementary Table 2. Calculated %*V*_Bur of MOAr and $M(OAr)_4$ using SAMBVCA 2.0 for a sphere of 3.5 Å radius centred on the metal centre with <MOC = 180°.

Metal (M)	MOAr %*V*_Bur	$M(OAr)_4$ %*V*_Bur
Zr	27.4	109.6
Ce	24.4	97.6
U	24.4	97.6
Th	23.4	93.6

1.15 Comparison of the estimated %*V*_Bur above with those calculated for $U(OAr)_4$ for CDS structure 1185610.

A number of approximations were used for estimating the %*V*_Bur given in Supplementary Table 2 for the ligands in $M(OAr)_4$ (M = Zr, Ce, Th, U), including using estimates of M-OAr bond length from the CSD v5.40 for the mean M-O bond length for tetraaryloxide complexes of these metals, and using a MOC angle of 180°. For comparison, the Th-OAr and U-OAr bond lengths observed in their $M(OAr)_4$ structures are 2.189(4) Å and 2.135(5) Å, respectively, while the respective mean Th-OAr and U-OAr bond lengths in the CSD for thorium and uranium tetra-coordinate aryloxide complexes are 2.192 Å and 2.119 Å.

For comparison, the %*V*_Bur of $U(OAr)_4$ was calculated for the structure given in CSD structure 1185610 (UOC angles = 154.0, U-OAr bond distances 2.135Å), for $U(OAr)_4$ with UOC angles of 154.0° and U-OAr bond lengths of 2.119 Å (the CSD average), and for $U(OAr)_4$ with UOC angles of 180° and U-OAr bond lengths of 2.135Å (see Supplementary Table 3 below).

Supplementary Table 3. Comparison of the %*V*_Bur for $U(OAr)_4$ complexes, with varying bond length and MOC angle. The row marked with a * indicates that the coordinates were taken directly from the reported $U(OAr)_4$ structure (CSD structure 1185610).

U-O-C angle (°)	U-O bond length (Å)	MOAr %*V*_Bur	$M(OAr)_4$ %*V*_Bur
154.0	2.119	24.7	98.8
154.0*	2.135*	24.5	98.0
180.0	2.119	24.4	97.6
180.0	2.135	24.2	96.8

The estimates of %*V*_Bur for varying UOC angles show that artificially fixing the MOC angle to 180° decreases the %*V*_Bur of the ligand in comparison with when %*V*_Bur is calculated for the bent MOC angle observed in the $U(OAr)_4$ complex for both MO bond lengths. This means that our estimates for %*V*_Bur are unlikely to be overestimates at the chosen bond lengths for the series of $M(OAr)_4$ calculated in Supplementary Table 2. However, using the mean M-O tetraaryloxide bond lengths found from the CDS, may result in an overestimation of the %*V*_Bur of the ligand as bulky metal(IV) aryloxides can form elongated bonds to accommodate the coordination of bulky ligands (see $U(OAr)_4$).[13] As shown in Supplementary Table 3, even minor changes in the metal-oxygen bond length significantly impact the calculated %*V*_Bur: an increase of

only 0.016 Å in the U-OAr bond length from the CSD mean of 2.119 Å, to that observed for $U(OAr)_4$ at 2.135(4) Å, is sufficient to reduce the total %*V*_Bur for four OAr ligands from 97.6 to 96.8% (when the UOC angle is also set to 180°).

Given the ease of preparation of $U(OAr)_4$ and the similar ionic radii of cerium(IV) and uranium(IV), at first glance it seemed that it should be possible to prepare $Ce(OAr)_4$ but the reduction potentials of Ce(IV) versus U(IV) explain why $Ce(OAr)_3$ is observed (Supplementary Table 4). The $U(OAr)_4$ complex is particularly crowded, resulting in U-O bond lengths longer than typically found for tetra-aryloxide complexes of uranium(IV). In the product, or presumed reaction intermediates $Ce(N^iPr_2)_x(OAr_3)_{4-x}$, this crowding is relieved by reductive Ce-X bond homolysis (X = N or O), resulting in the $Ce(III)OAr_3$. N.b. it is unclear whether it is $^{\cdot}N^iPr_2$ or $^{\cdot}OAr$ that is eliminated by bond homolysis as the expected coupled products are not observed in ^1H-NMR spectra and products arising from H abstraction from solvent would be indistinguishable (we do not observe the H in HN^iPr_2). For reference the %*V*Bur of N^iPr_2 is 21.9 % ($Ce(N^iPr_2)_4$ total %*V*_bur = 87.6 %).

Supplementary Table 4: Reduction potentials of U(IV) and Ce(IV) with respect to a standard hydrogen electrode.

**Reaction**	***E*°/V** (with respect to standard hydrogen electrode)[14]
$U^{4+} + e^- \rightleftharpoons U^{3+}$	-0.52
$Ce^{4+} + e^- \rightleftharpoons Ce^{3+}$	+1.72

Zirconium(IV) has a significantly smaller ionic radius than cerium(IV) or uranium (IV), with typical Zr(IV) tetraaryloxide Zr-O bond lengths of 1.913 Å. On the basis of the estimated %V_Bur of 109.6% for $Zr(OAr)_4$ we do not expect it to be possible to form $Zr(OAr)_4$, which is in line with our own, and others', experience.[15]

1.16 Crystal Structure Determinations

Single crystals of each material were loaded into Merrill-Bassett diamond anvil cells[16, 17] with a chip of ruby to enable the pressure to be measured from its fluorescence wavelength. Hydrostatic media were fluorinert FC70 (0-1 GPa), pentane-isopentane (1-5 GPa) and Daphne oil (0-2.5 GPa). For the Th and U derivatives data were collected on beamline I19 at Diamond Light Source using radiation of wavelength 0.4859 Å. Additional data for $Th(OAr)_4$ at ambient pressure, 2.83, 3.16 and 4.30 GPa and for $U(OAr)_4$ at ambient pressure, 0.44, 1.84 and 2.67 GPa and all data for $Np(OAr)_4$ were measured using Mo *K*α radiation on Bruker Apex diffractometers with sealed-tube sources. Data were collected to maximum pressures of 4.3, 3.9 and 4.1 GPa for the Th, U and Np systems.

Increasing the pressure from 2.88 to 3.03 GPa for $Th(OAr)_4$ and from 2.88 to 3.02 GPa for $U(OAr)_4$ led to a degradation in the quality of the diffraction data as a result of the phase transitions at 3 GPa and possibly partial amorphisation, Supplementary Figure 5 showing the variation of R_{int} with resolution for both complexes at these pressures. Although the peaks showed some broadening, there was no sign of splitting of peaks, which might indicate a reduction in symmetry and twinning, but there was a marked decrease in the intensity of the high-resolution data (Supplementary Figure 6). Similar comments can be applied to the data for $U(OAr)_4$ at 2.88 and 3.02 GPa. The decline in crystal quality led to increases in the uncertainties of the structural parameters at the highest pressures achieved in this study.

For $Np(OAr)_4$ significant intensity was only available to atomic resolution up to 2.4 GPa as synchrotron radiation could not be used for this highly radioactive sample. Above 2.4 GPa only unit cell dimensions could be determined: a = 12.6756(12) and c = 13.066(3) Å at 3.3 GPa and a = 12.5791(12) and c = 12.963(3) Å at 4.1 GPa.

Crystal structures were solved using SHELXT[18] and refined against $|F|^2$. Distances and angles within the ligands restrained in the all the refinements against high-pressure data to ambient pressure values; enhanced rigid body restraints were also applied to the anisotropic displacement parameters. Equation of state analysis was

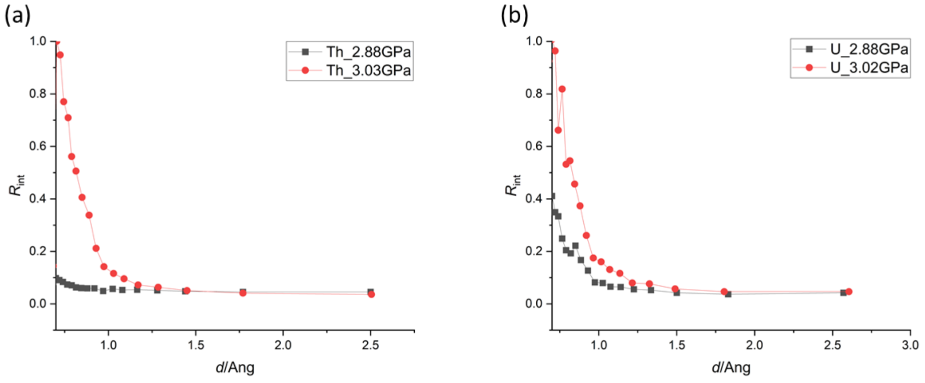

Supplementary Figure 5. Variation of the merging R factor (R_{int}) for the data sets collected for (a) $Th(OAr)_4$ and (b) $U(OAr)_4$ immediately above and below the phase transition at *ca.* 3 GPa.

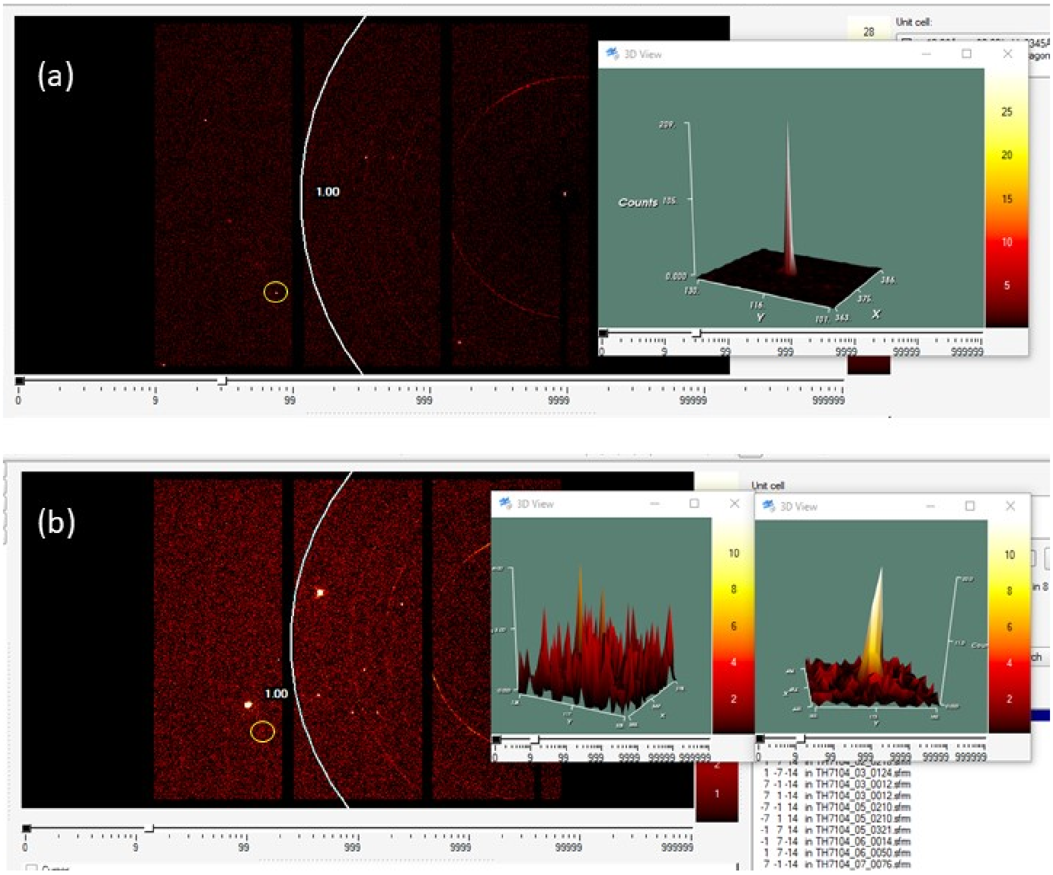

Supplementary Figure 6: Diffraction images of $Th(OAr)_4$ above and below the phase transition at 3 GPa. (a) Image at 2.88 GPa showing the profile of the 1 −7 −14 reflection, selected arbitrarily and circled in yellow. (b) Profile of the region where the same reflection would be expected at 3.03 GPa. Also shown is the profile of a lower-resolution reflection showing the lack of splitting.

carried out with EoSFIT.[19] Crystallographic information files are available in the Cambridge Database with deposition numbers 1981193-1981195, 1981197-1981201, 1981204-1981216 and 2095929-2095947. Crystal and refinement data are also listed in Supplementary Tables 6, 7 and 8.

1.17 Projection analysis of crystallographic data

In order to identify the structural changes that give rise to the variation in unit cell dimensions, the dimensions of different fragments of the $M(OAr)_4$ crystal structures projected onto the *a* and *c* axes were calculated. The dimensions of the MO_4 moiety probe the immediate coordination environment of the metals; the difference between the $M(C1)_4$ dimensions and those of the MO_4 moiety is the length of a 'link' region which probes the role of the M-O-C1 angle (C1 = the ipso C-atom of the phenyl ring). The difference between the $M(C1)_4$ dimensions and the lengths of the *a* and *c* axes probes the role of the remainder of the complex and the intermolecular interactions. The following example illustrate the calculation:

The length (or 'height') of the ThO_4 tetrahedron in $Th(OAr)_4$ projected onto **c** at ambient pressure.

The cell dimensions are *a* = 14.0696(2), *c* = 13.6511(2) Å and the coordinates of the Th, O1 and C1 atoms are:

	x	σ(*x*)	*y*	σ (*y*)	*z*	σ (z)
Th1	0	0	½	0	¼	0
O1	0.0872	0.00012	0.41106	0.00012	0.34573	0.00012
C1	0.1125	0.0004	0.3669	0.0004	0.4307	0.0004

The contribution of lengths of the MO_4 pseudo-tetrahedra projected onto *c* is $4|z(O1) - z(Th1)|c$, the '4' arising because the difference $z(O1) - z(Th1)$ measures the half height of the tetrahedron and that there are two molecules of ML_4 per unit cell:

2 x 2 x 13.6511 x (0.34573 – ¼) = 5.2273 Å

The variance is given by $16[c^2\sigma^2(z(O1)) + (z(O1) - z(O1))^2\sigma^2(c)$, giving a standard uncertainty of 0.0066 Å.

The projected dimension of the $Th(C1)_4$ moiety along **c** is calculated in the same way, giving 9.867(22) Å, so that the contribution of the 'link' regions to *c* is 9.867(22) – 5.2273(66) = 4.640(23) Å.

The contribution of the remainder of the structure is obtained in this example from the difference of these two measurements with the length of the *c* axis: 13.6511(2) – 9.867(22) = 3.784(22).

A similar calculation can be carried out along the *a* axis using the midpoint of O1 and O1b to define the dimension of the ThO_4 tetrahedron (see Fig. 1 in the main paper, where O1b is generated by the operation ½ − y, ½ + *x*, ½ − *z*). The advantage of using the midpoint is that the breakdown of contributions is the same along the *a* and *b* axes. The dimension of the $Th(C1)_4$ and thereby the 'link' contribution, is likewise defined using the midpoint between C1 and C1b.

Plots showing the variation of the projected dimensions with pressure are shown in Supplementary Figure 9. The important point is that collapse in the *c* axis length and the slight increase in the *a* axis length at 3 GPa for $Th(OAr)_4$ and $U(OAr)_4$ arises from the discontinuous changes in the dimensions of ThO_4 and UO_4 tetrahedra projected along **c** and **a**, respectively.

1.18 Shape index calculations

The continuous symmetry measure, or shape index, is a parameter devised originally by Pinksy and Avnir[20] to assess the extent of distortion of a given polyhedron from an ideal reference polyhedron.[21] In the context of the present study, the parameter was used to measure how far the MO_4 moiety in $M(OAr)_4$ (M = Th, U or Np) deviated from an ideal tetrahedron as a function of pressure; a shape index of zero corresponds to an ideal tetrahedron.

The atomic coordinates of the M and O atoms contained in the cifs (see above) were converted from fractional to Cartesian using Mercury and then used as input to the program SHAPE.[22] The following is a fragment of the input file for $Th(OAr)_4$, where the geometries of the ThO_4 unit at ambient pressure and 4.30 GPa is compared with both a tetrahedron and a square; see Example 1 in the SHAPE manual for further explanatory notes.

```
$ ThL4 structures
! Ligands  Metal
     4       1
! Tetrahedron Square
     2         1
Th(OAr4)_0_GPa
Th       0.000000    7.034800    3.412775
O        1.226869    5.783450    4.719595
O       -1.251350    5.807931    2.105955
O       -1.226869    8.286150    4.719595
O        1.251350    8.261669    2.105955
```

The shape index for the complex at 0 GPa is 0.064 relative to a tetrahedron and 32.180 relative to a square, meaning that it closely resembles a tetrahedron, but is very unlike a square. Standard uncertainties are not calculated by the SHAPE software. The shape indices for MO_4 moieties in $Th(OAr)_4$, $U(OAr)_4$ and $Np(OAr)_4$ as a function of pressure are plotted in Supplementary Figure 11.

2: Supplementary Tables

Supplementary Table 5: Selected geometric parameters for $M(OAr)_4$, (M = Th, U, Np) at ambient conditions.

	Th	U	Np
T / *K*	270	270	253
V_0	2702.29(9)	2691.17(17)	2669.9(1.5)
a_0	14.0696(2)	14.0749(4)	14.041(3)
c_0	13.6511(2)	13.5847(4)	13.542(4)
$a(O_{1a}\text{-}M\text{-}O_{1b})$	106.58(6)	106.22(7)	105.4(3)
$a(O_{1a}\text{-}M\text{-}O_{1c})$	110.94(6)	111.12(7)	111.6(3)
$d(M\text{-}O_{1a})$	2.1861(17)	2.1453(19)	2.129(7)
$a(M\text{-}O_1\text{-}C_1)$	156.4(3)	156.7(2)	156.8(12)
$t(M\text{-}O_{1a}\text{-}C_1\text{-}C_2)$	-109.6(6)	-111.6(6)	-108(3)
$t(C_1\text{-}C_2\text{-}C_7\text{-}C_{10})$	-172.3(4)	-172.1(3)	-172.2(17)
$t(C_1\text{-}C_6\text{-}C_{11}\text{-}C_{14})$	-22.7(6)	-20.7(6)	-20(3)

Supplementary Table 6. Crystal and refinement data for $Th(OAr)_4$

For all structures: $C_{56}H_{84}O_4Th$, M_r = 1053.32, Tetragonal, $I\bar{4}$, Z = 2. Experiments were carried out at 293 K.

Pressure (GPa)	Ambient	0.26	0.52	0.58
Crystal data				
a, c (Å)	14.0696 (2), 13.6511 (2)	13.8282 (2), 13.5879 (5)	13.6824 (3), 13.5649 (3)	13.6477 (4), 13.5258 (18)
V (Å^3)	2702.29 (9)	2598.27 (12)	2539.46 (12)	2519.3 (4)
Radiation type	Mo $K\alpha$	Synchrotron, λ = 0.48590 Å	Synchrotron, λ = 0.48590 Å	Synchrotron, λ = 0.48590 Å
μ (mm^{-1})	2.80	1.76	1.80	1.81
Crystal size (mm)	0.30 × 0.20 × 0.20	0.20 × 0.15 × 0.10	0.20 × 0.15 × 0.15	0.20 × 0.15 × 0.10
Data collection				
Diffractometer	Bruker Apex2	Diamond I19-EH2	Diamond I19-EH2	Diamond I19-EH2
Absorption correction	Multi-scan *SADABS* (Siemens, 1996)	Multi-scan *AIMLESS* (CCP4, 2018)	Multi-scan *AIMLESS* (CCP4, 2018)	Multi-scan *AIMLESS* (CCP4, 2018)
T_{min}, T_{max}	0.43, 0.57	0.988, 1.0	0.989, 1.0	0.998, 1.0
No. of measured, independent and observed [$I > 2.0\sigma(I)$] reflections	12691, 3188, 3179	8180, 3322, 3197	5375, 2327, 2305	3564, 2439, 2012
R_{int}	0.024	0.055	0.038	0.042
$(\sin \theta/\lambda)_{max}$ (Å^{-1})	0.666	0.786	0.788	0.778
Refinement				
$R[F^2 > 2\sigma(F^2)]$, $wR(F^2)$, S	0.018, 0.017, 1.51	0.032, 0.034, 0.98	0.029, 0.029, 1.03	0.037, 0.039, 0.91
No. of reflections	3179	3197	2305	2012
No. of parameters	139	139	139	139
No. of restraints	45	84	84	84
H-atom treatment	H-atom parameters constrained	H-atom parameters not refined	H-atom parameters not refined	H-atom parameters not refined
$\Delta\rho_{max}$, $\Delta\rho_{min}$ (e Å^{-3})	0.19, -0.09	0.22, -0.43	0.75, -0.55	0.44, -0.33
Absolute structure	Parsons, Flack & Wagner (2013), 1479 Friedel Pairs	Parsons, Flack & Wagner (2013), 1484 Friedel Pairs	Parsons, Flack & Wagner (2013), 788 Friedel Pairs	Parsons, Flack & Wagner (2013), 745 Friedel Pairs
Absolute structure parameter	0.710 (4)	0.499 (16)	1.008 (17)	0.99 (3)

Supplementary Table 6 Continued

Pressure (GPa)	0.91	1.37	1.77	2.04
Crystal data				
a, *c* (Å)	13.4289 (3), 13.5510 (3)	13.2089 (4), 13.5581 (3)	13.0469 (3), 13.5793 (3)	12.9516 (3), 13.5965 (3)
V ($Å^3$)	2443.72 (12)	2365.55 (14)	2311.49 (11)	2280.73 (11)
Radiation type	Synchrotron, λ = 0.48590 Å	Synchrotron, λ = 0.48590 Å	Synchrotron, λ = 0.48590 Å	Synchrotron, λ = 0.48590 Å
μ (mm^{-1})	1.87	1.93	1.97	2.00
Crystal size (mm)	0.20 × 0.15 × 0.15	0.20 × 0.15 × 0.15	0.20 × 0.15 × 0.15	0.20 × 0.15 × 0.15
Data collection				
Diffractometer	Diamond I19-EH2	Diamond I19-EH2	Diamond I19-EH2	Diamond I19-EH2
Absorption correction	Multi-scan *AIMLESS* (CCP4, 2018)	Multi-scan *AIMLESS* (CCP4, 2018)	Multi-scan *AIMLESS* (CCP4, 2018)	Multi-scan *AIMLESS* (CCP4, 2018)
T_{min}, T_{max}	0.989, 1.0	0.989, 1.0	0.991, 1.0	0.992, 1.0
No. of measured, independent and observed [$I > 2.0\sigma(I)$] reflections	5165, 2233, 2220	4991, 2152, 2056	4875, 2113, 2056	4801, 2081, 2055
R_{int}	0.036	0.036	0.034	0.034
$(\sin \theta/\lambda)_{max}$ ($Å^{-1}$)	0.788	0.792	0.794	0.792
Refinement				
$R[F^2 > 2\sigma(F^2)]$, $wR(F^2)$, S	0.030, 0.031, 1.00	0.033, 0.032, 1.00	0.030, 0.030, 1.00	0.031, 0.029, 1.00
No. of reflections	2220	2056	2056	2055
No. of parameters	139	139	139	139
No. of restraints	84	84	84	84
H-atom treatment	H-atom parameters constrained	H-atom parameters constrained	H-atom parameters constrained	H-atom parameters not refined
$\Delta\rho_{max}$, $\Delta\rho_{min}$ (e $Å^{-3}$)	0.47, -0.43	0.74, -0.72	0.51, -0.35	0.64, -0.31
Absolute structure	Parsons, Flack & Wagner (2013), 763 Friedel Pairs	Parsons, Flack & Wagner (2013), 709 Friedel Pairs	Parsons, Flack & Wagner (2013), 706 Friedel Pairs	Parsons, Flack & Wagner (2013), 707 Friedel Pairs
Absolute structure parameter	0.999 (16)	0.001 (17)	-0.005 (16)	0.998 (15)

Supplementary Table 6 Continued

Pressure (GPa)	2.41	2.44	2.62	2.83
Crystal data				
a, *c* (Å)	12.7987 (4), 13.6509 (5)	12.8099 (3), 13.6621 (4)	12.7617 (2), 13.6588 (3)	12.6948 (5), 13.6644 (7)
V ($Å^3$)	2236.11 (17)	2241.86 (12)	2224.49 (9)	2202.1 (2)
Radiation type	Synchrotron, λ = 0.48590 Å	Synchrotron, λ = 0.48590 Å	Synchrotron, λ = 0.48590 Å	Mo *K*α
μ (mm^{-1})	2.04	2.04	2.05	3.44
Crystal size (mm)	0.20 × 0.15 × 0.15	0.20 × 0.15 × 0.10	0.20 × 0.15 × 0.10	0.20 × 0.15 × 0.10
Data collection				
Diffractometer	Diamond I19-EH2	Diamond I19-EH2	Diamond I19-EH2	Bruker Apex2
Absorption correction	Multi-scan *AIMLESS* (CCP4, 2018)	Multi-scan *AIMLESS* (CCP4, 2018)	Multi-scan *AIMLESS* (CCP4, 2018)	Multi-scan *SADABS* (Siemens, 1996)
T_{min}, T_{max}	0.991, 1.0	0.997,1.0	0.996, 1.0	0.60, 0.71
No. of measured, independent and observed [$I > 2.0\sigma(I)$] reflections	4486, 1990, 1840	5636, 2754, 2744	7133, 2971, 2948	3157, 1420, 1368
R_{int}	0.033	0.049	0.047	0.038
$(\sin \theta/\lambda)_{max}$ ($Å^{-1}$)	0.791	0.797	0.797	0.647
Refinement				
$R[F^2 > 2\sigma(F^2)]$, $wR(F^2)$, S	0.033, 0.029, 1.00	0.035, 0.033, 0.99	0.029, 0.028, 0.96	0.035, 0.037, 1.00
No. of reflections	1840	2744	2948	1368
No. of parameters	139	139	139	139
No. of restraints	84	84	84	84
H-atom treatment	H-atom parameters not refined	H-atom parameters not refined	H-atom parameters constrained	H-atom parameters not refined
$\Delta\rho_{max}$, $\Delta\rho_{min}$ (e $Å^{-3}$)	0.87, -0.62	0.43, -0.56	0.20, -0.33	0.35, -0.72
Absolute structure	Parsons, Flack & Wagner (2013), 623 Friedel Pairs	Parsons, Flack & Wagner (2013), 1179 Friedel Pairs	Parsons, Flack & Wagner (2013), 1390 Friedel Pairs	Parsons, Flack & Wagner (2013), 632 Friedel Pairs
Absolute structure parameter	-0.003 (16)	0.434 (14)	0.458 (12)	0.938 (15)

Supplementary Table 6 Continued

Pressure (GPa)	2.88	3.03	3.16	3.42
Crystal data				
a, *c* (Å)	12.6900 (2), 13.6517 (3)	12.8178 (4), 13.1943 (5)	12.8022 (8), 13.1861 (8)	12.7582 (4), 13.1398 (5)
V (Å^3)	2198.42 (9)	2167.77 (16)	2161.2 (3)	2138.79 (14)
Radiation type	Synchrotron, λ = 0.48590 Å	Synchrotron, λ = 0.48590 Å	Mo $K\alpha$	Synchrotron, λ = 0.48590 Å
μ (mm^{-1})	2.08	2.10	3.50	2.13
Crystal size (mm)	0.20 × 0.15 × 0.10	0.20 × 0.15 × 0.10	0.20 × 0.15 × 0.10	0.20 × 0.15 × 0.10
Data collection				
Diffractometer	Diamond I19-EH2	Diamond I19-EH2	Bruker Apex2	Diamond I19-EH2
Absorption correction	Multi-scan *AIMLESS* (CCP4, 2018)	Multi-scan *AIMLESS* (CCP4, 2018)	Multi-scan *SADABS* (Siemens, 1996)	Multi-scan *AIMLESS* (CCP4, 2018)
T_{min}, T_{max}	0.996, 1.0	0.998, 1.0	0.59, 0.70	0.999, 1.0
No. of measured, independent and observed [$I > 2.0\sigma(I)$] reflections	7048, 2928, 2911	6896, 2837, 1307	4302, 1462, 935	6802, 2825, 1188
R_{int}	0.049	0.048	0.051	0.045
$(\sin \theta/\lambda)_{max}$ (Å^{-1})	0.798	0.682	0.617	0.671
Refinement				
$R[F^2 > 2\sigma(F^2)]$, $wR(F^2)$, S	0.029, 0.028, 0.95	0.092, 0.066, 0.99	0.089, 0.065, 1.07	0.084, 0.054, 0.96
No. of reflections	2911	1307	934	1188
No. of parameters	139	139	139	139
No. of restraints	84	84	120	84
H-atom treatment	H-atom parameters constrained	H-atom parameters constrained	H-atom parameters not refined	H-atom parameters constrained
$\Delta\rho_{max}$, $\Delta\rho_{min}$ (e Å^{-3})	0.21, -0.36	0.38, -0.35	0.47, -0.26	0.31, -0.29
Absolute structure	Parsons, Flack & Wagner (2013), 1373 Friedel Pairs	Parsons, Flack & Wagner (2013), 545 Friedel Pairs	Parsons, Flack & Wagner (2013), 380 Friedel Pairs	Parsons, Flack & Wagner (2013), 485 Friedel Pairs
Absolute structure parameter	0.449 (12)	0.565 (16)	0.118 (17)	0.554 (16)

Supplementary Table 6 Continued

Pressure (GPa)	4.30
Crystal data	
a, *c* (Å)	12.6296 (12), 13.0316 (11)
V ($Å^3$)	2078.6 (4)
Radiation type	Mo *K*α
μ (mm^{-1})	3.64
Crystal size (mm)	0.20 × 0.15 × 0.10
Data collection	
Diffractometer	Bruker Apex2
Absorption correction	Multi-scan *SADABS* (Siemens, 1996)
T_{min}, T_{max}	0.58, 0.69
No. of measured, independent and observed [$I > 2.0\sigma(I)$] reflections	4020, 1381, 731
R_{int}	0.064
$(\sin \theta/\lambda)_{max}$ ($Å^{-1}$)	0.595
Refinement	
$R[F^2 > 2\sigma(F^2)]$, $wR(F^2)$, S	0.100, 0.066, 1.11
No. of reflections	731
No. of parameters	137
No. of restraints	120
H-atom treatment	H-atom parameters not refined
$\Delta\rho_{max}$, $\Delta\rho_{min}$ (e $Å^{-3}$)	0.31, -0.20
Absolute structure	Parsons, Flack & Wagner (2013), 281 Friedel Pairs
Absolute structure parameter	0.87 (2)

Supplementary Table 7. Crystal and refinement data for U(OAr)4

For all structures: $C_{56}H_{84}O_4U$, M_r = 1059.31, Tetragonal, $I\bar{4}$, Z = 2. Experiments were carried out at 293 K.

Pressure (GPa)	ambient	0.14	0.30	0.44
Crystal data				
a, c (Å)	14.0749 (4), 13.5847 (4)	13.9245 (4), 13.5337 (6)	13.7957 (4), 13.4995 (5)	13.7071 (4), 13.5020 (4)
V (Å^3)	2691.17 (17)	2624.07 (18)	2569.24 (18)	2536.82 (17)
Radiation type	Mo $K\alpha$	Synchrotron, λ = 0.48590 Å	Synchrotron, λ = 0.48590 Å	Mo $K\alpha$
μ (mm^{-1})	3.06	1.88	1.92	3.24
Crystal size (mm)	0.30 × 0.25 × 0.20	0.20 × 0.15 × 0.10	0.20 × 0.15 × 0.10	0.20 × 0.15 × 0.10
Data collection				
Diffractometer	Bruker Apex2	Diamond I19-EH2	Diamond I19-EH2	Diamond I19-EH2
Absorption correction	Multi-scan *SADABS* (Siemens, 1996)	Multi-scan *AIMLESS* (CCP4, 2018)	Multi-scan *AIMLESS* (CCP4, 2018)	Multi-scan *SADABS* (Siemens, 1996)
T_{min}, T_{max}	0.47, 0.54	0.998, 1.0	0.997, 1.0	0.61, 0.72
No. of measured, independent and observed [$I > 2.0\sigma(I)$] reflections	12279, 3186, 3170	3786, 2393, 1921	3703, 2324, 1508	4931, 1642, 1592
R_{int}	0.033	0.088	0.091	0.039
$(\sin \theta/\lambda)_{max}$ (Å^{-1})	0.669	0.767	0.761	0.643
Refinement				
$R[F^2 > 2\sigma(F^2)]$, $wR(F^2)$, S	0.024, 0.019, 1.00	0.048, 0.048, 1.00	0.043, 0.045, 1.00	0.031, 0.022, 1.46
No. of reflections	3170	1921	1508	1592
No. of parameters	139	139	139	139
No. of restraints	45	84	84	84
H-atom treatment	H-atom parameters constrained	H-atom parameters constrained	H-atom parameters constrained	H-atom parameters constrained
$\Delta\rho_{max}$, $\Delta\rho_{min}$ (e Å^{-3})	0.20, -0.17	0.66, -0.65	0.47, -0.62	0.16, -0.16
Absolute structure	Parsons, Flack & Wagner (2013), 1465 Friedel Pairs	Parsons, Flack & Wagner (2013), 712 Friedel Pairs	Parsons, Flack & Wagner (2013), 545 Friedel Pairs	Parsons, Flack & Wagner (2013), 735 Friedel Pairs
Absolute structure parameter	1.025 (3)	0.83 (4)	0.89 (4)	-0.008 (6)

Supplementary Table 7 Continued

Pressure (GPa)	0.56	0.70	1.11	1.84
Crystal data				
a, *c* (Å)	13.6436 (3), 13.4745 (8)	13.5572 (4), 13.4550 (5)	13.3367 (4), 13.4817 (11)	13.0495 (11), 13.5258 (18)
V ($Å^3$)	2508.25 (18)	2473.00 (16)	2398.0 (2)	2303.3 (5)
Radiation type	Synchrotron, λ = 0.48590 Å	Synchrotron, λ = 0.48590 Å	Synchrotron, λ = 0.48590 Å	Mo *K*α
μ (mm^{-1})	1.97	2.00	2.06	3.57
Crystal size (mm)	0.20 × 0.15 × 0.15	0.20 × 0.15 × 0.10	0.20 × 0.15 × 0.15	0.20 × 0.15 × 0.10
Data collection				
Diffractometer	Diamond I19-EH2	Diamond I19-EH2	Diamond I19-EH2	Bruker Apex2
Absorption correction	Multi-scan *AIMLESS* (CCP4, 2018)	Multi-scan *AIMLESS* (CCP4, 2018)	Multi-scan *AIMLESS* (CCP4, 2018)	Multi-scan *SADABS* (Siemens, 1996)
T_{min}, T_{max}	0.982, 1.0	0.996, 1.0	0.996, 1.0	0.59, 0.70
No. of measured, independent and observed [$I > 2.0\sigma(I)$] reflections	5251, 2843, 2685	3507, 2217, 1550	4768, 2635, 2398	3991, 1632, 1485
R_{int}	0.044	0.086	0.035	0.037
$(\sin \theta/\lambda)_{max}$ ($Å^{-1}$)	0.787	0.751	0.784	0.644
Refinement				
$R[F^2 > 2\sigma(F^2)]$, $wR(F^2)$, S	0.033, 0.030, 0.83	0.044, 0.046, 1.00	0.037, 0.035, 0.99	0.046, 0.042, 1.00
No. of reflections	2685	1550	2398	1485
No. of parameters	139	138	139	139
No. of restraints	84	84	84	84
H-atom treatment	H-atom parameters constrained	H-atom parameters not refined	H-atom parameters constrained	H-atom parameters constrained
$\Delta\rho_{max}$, $\Delta\rho_{min}$ (e $Å^{-3}$)	0.58, -0.43	0.58, -0.71	1.05, -1.03	0.24, -0.39
Absolute structure	Parsons, Flack & Wagner (2013), 934 Friedel Pairs	Parsons, Flack & Wagner (2013), 557 Friedel Pairs	Parsons, Flack & Wagner (2013), 821 Friedel Pairs	Parsons, Flack & Wagner (2013), 670 Friedel Pairs
Absolute structure parameter	0.009 (17)	0.12 (4)	0.998 (16)	0.865 (12)

Supplementary Table 7 Continued

Pressure (GPa)	2.37	2.47	2.67	2.67 (repeat)
Crystal data				
a, *c* (Å)	12.8313 (3), 13.5678 (10)	12.8046 (2), 13.5695 (10)	12.7417 (2), 13.5729 (10)	12.7501 (7), 13.6186 (11)
V (Å^3)	2233.8 (2)	2224.83 (18)	2203.57 (18)	2213.9 (3)
Radiation type	Synchrotron, λ = 0.48590 Å	Synchrotron, λ = 0.48590 Å	Synchrotron, λ = 0.48590 Å	Mo $K\alpha$
μ (mm^{-1})	2.21	2.22	2.24	3.72
Crystal size (mm)	0.20 × 0.15 × 0.10	0.20 × 0.15 × 0.10	0.20 × 0.15 × 0.10	0.20 × 0.15 × 0.10
Data collection				
Diffractometer	Diamond I19-EH2	Diamond I19-EH2	Diamond I19-EH2	Bruker Apex2
Absorption correction	Multi-scan *AIMLESS* (CCP4, 2018)	Multi-scan *AIMLESS* (CCP4, 2018)	Multi-scan *AIMLESS* (CCP4, 2018)	Multi-scan *SADABS* (Siemens, 1996)
T_{min}, T_{max}	0.998, 1.0	0.997, 1.0	0.997, 1.0	0.57, 0.69
No. of measured, independent and observed [$I > 2.0\sigma(I)$] reflections	6971, 2441, 2317	6993, 2438, 2338	6911, 2416, 2342	3776, 1581, 1411
R_{int}	0.052	0.051	0.047	0.045
$(\sin \theta/\lambda)_{max}$ (Å^{-1})	0.788	0.789	0.784	0.642
Refinement				
$R[F^2 > 2\sigma(F^2)]$, $wR(F^2)$, S	0.035, 0.032, 1.07	0.033, 0.031, 1.05	0.032, 0.030, 1.00	0.049, 0.044, 1.01
No. of reflections	2317	2338	2342	1411
No. of parameters	139	139	139	139
No. of restraints	84	84	84	84
H-atom treatment	H-atom parameters constrained	H-atom parameters constrained	H-atom parameters constrained	H-atom parameters constrained
$\Delta\rho_{max}$, $\Delta\rho_{min}$ (e Å^{-3})	0.22, -0.23	0.18, -0.28	0.28, -0.22	0.22, -0.26
Absolute structure	Parsons, Flack & Wagner (2013), 1043 Friedel Pairs	Parsons, Flack & Wagner (2013), 1059 Friedel Pairs	Parsons, Flack & Wagner (2013), 1070 Friedel Pairs	Parsons, Flack & Wagner (2013), 649 Friedel Pairs
Absolute structure parameter	0.814 (13)	0.193 (13)	0.207 (12)	0.843 (13)

Supplementary Table 7 Continued

Pressure (GPa)	2.85	2.88	3.02	3.32
Crystal data				
a, *c* (Å)	12.7090 (2), 13.5665 (10)	12.7237 (3), 13.5250 (14)	12.7703 (4), 13.2793 (17)	12.7424 (2), 13.1705 (10)
V ($Å^3$)	2191.24 (18)	2189.6 (2)	2165.6 (3)	2138.48 (18)
Radiation type	Synchrotron, λ = 0.48590 Å	Synchrotron, λ = 0.48590 Å	Synchrotron, λ = 0.48590 Å	Synchrotron, λ = 0.48590 Å
μ (mm^{-1})	2.26	2.26	2.28	2.31
Crystal size (mm)	0.20 × 0.15 × 0.10	0.20 × 0.15 × 0.10	0.20 × 0.15 × 0.10	0.20 × 0.15 × 0.10
Data collection				
Diffractometer	Diamond I19-EH2	Diamond I19-EH2	Diamond I19-EH2	Diamond I19-EH2
Absorption correction	Multi-scan *AIMLESS* (CCP4, 2018)	Multi-scan *AIMLESS* (CCP4, 2018)	Multi-scan *AIMLESS* (CCP4, 2018)	Multi-scan *AIMLESS* (CCP4, 2018)
T_{min}, T_{max}	0.998, 1.0	0.997, 1.0	0.997, 1.0	0.998, 1.0
No. of measured, independent and observed [$I > 2.0\sigma(I)$] reflections	6774, 2392, 2297	6403, 2299, 1906	6506, 2329, 1193	6673, 2363, 1190
R_{int}	0.049	0.054	0.065	0.048
$(\sin \theta/\lambda)_{max}$ ($Å^{-1}$)	0.783	0.765	0.672	0.706
Refinement				
$R[F^2 > 2\sigma(F^2)]$, $wR(F^2)$, S	0.036, 0.032, 1.00	0.047, 0.036, 1.00	0.078, 0.061, 0.99	0.086, 0.065, 1.00
No. of reflections	2297	1906	1193	1190
No. of parameters	139	139	139	139
No. of restraints	84	84	84	84
H-atom treatment	H-atom parameters constrained	H-atom parameters constrained	H-atom parameters constrained	H-atom parameters constrained
$\Delta\rho_{max}$, $\Delta\rho_{min}$ (e $Å^{-3}$)	0.31, -0.28	0.22, -0.24	0.33, -0.50	0.31, -0.26
Absolute structure	Parsons, Flack & Wagner (2013), 1042 Friedel Pairs	Parsons, Flack & Wagner (2013), 831 Friedel Pairs	Parsons, Flack & Wagner (2013), 504 Friedel Pairs	Parsons, Flack & Wagner (2013), 493 Friedel Pairs
Absolute structure parameter	0.204 (12)	0.214 (14)	0.77 (2)	0.707 (15)

Supplementary Table 7 Continued

Pressure (GPa)	3.88
Crystal data	
a, *c* (Å)	12.6604 (4), 13.0759 (15)
V ($Å^3$)	2095.9 (3)
Radiation type	Synchrotron, λ = 0.48590 Å
μ (mm^{-1})	2.36
Crystal size (mm)	0.20 × 0.15 × 0.10
Data collection	
Diffractometer	Diamond I19-EH2
Absorption correction	Multi-scan *AIMLESS* (CCP4, 2018)
T_{min}, T_{max}	0.998, 1.0
No. of measured, independent and observed [$I > 2.0\sigma(I)$] reflections	6451, 2310, 996
R_{int}	0.045
$(\sin \theta/\lambda)_{max}$ ($Å^{-1}$)	0.733
Refinement	
$R[F^2 > 2\sigma(F^2)]$, $wR(F^2)$, S	0.091, 0.070, 0.99
No. of reflections	996
No. of parameters	139
No. of restraints	84
H-atom treatment	H-atom parameters constrained
$\Delta\rho_{max}$, $\Delta\rho_{min}$ (e $Å^{-3}$)	0.27, -0.26
Absolute structure	Parsons, Flack & Wagner (2013), 400 Friedel Pairs
Absolute structure parameter	0.292 (17)

Supplementary Table 8. Crystal and refinement data for Np(OAr)$_4$

For all structures: $C_{56}H_{84}NpO_4$, M_r = 1058.23. Tetragonal, $I\bar{4}$, Z = 2. Experiments were carried out with Mo $K\alpha$ radiation. Absorption was corrected for by multi-scan methods, *SADABS* (Siemens, 1996).

Pressure (GPa)	Ambient	0.54	1.00	1.55
Crystal data				
Temperature (K)	253	293	293	293
a, c (Å)	14.041 (3), 13.542 (4)	13.5526 (10), 13.413 (2)	13.2750 (8), 13.3894 (14)	13.1192 (11), 13.383 (2)
V (Å^3)	2669.9 (15)	2463.7 (6)	2359.6 (4)	2303.3 (5)
μ (mm^{-1})	1.99	2.15	2.25	2.30
Crystal size (mm)	0.12 × 0.08 × 0.03	0.10 × 0.08 × 0.05	0.10 × 0.08 × 0.05	0.10 × 0.08 × 0.05
Data collection				
Diffractometer	Bruker Kappa Apex2	Bruker Kappa Apex2	Bruker Kappa Apex2	Bruker Kappa Apex2
T_{min}, T_{max}	0.89, 0.96	0.81, 0.90	0.89, 0.89	0.30, 0.89
No. of measured, independent and observed reflections	12143, 3247, 2368 [$I > 2\sigma(I)$]	1665, 1045, 854 [$I > 2.0\sigma(I)$]	2126, 1110, 925 [$I > 2.0\sigma(I)$]	1244, 1097, 798 [$I > 2.0\sigma(I)$]
R_{int}	0.123	0.059	0.050	0.033
θ_{max} (°)	28.8	23.2	23.3	26.4
$(\sin\theta/\lambda)_{max}$ (Å^{-1})	0.678	0.555	0.556	0.625
Refinement				
$R[F^2 > 2\sigma(F^2)]$, $wR(F^2)$, S	0.057, 0.104, 0.96	0.055, 0.045, 1.00	0.051, 0.042, 0.96	0.059, 0.044, 1.01
No. of reflections	3247	854	925	785
No. of parameters	144	139	139	139
No. of restraints	0	84	84	84
H-atom treatment	H-atom parameters constrained	H-atom parameters constrained	H-atom parameters not refined	H-atom parameters constrained
$\Delta\rho_{max}$, $\Delta\rho_{min}$ (e Å^{-3})	0.75, -0.62	0.29, -0.47	0.20, -0.16	0.73, -0.53
Absolute structure	Flack x determined using 791 quotients	Flack (1983), 448 Friedel pairs	Flack (1983), 549 Friedel pairs	Flack (1983), 434 Friedel pairs
Absolute structure parameter	0.45 (3)	0.44 (6)	0.59 (5)	0.47(6)

Supplementary Table 8 Continued

Pressure (GPa)	2.04	2.38
Crystal data		
Temperature (K)	293	293
a, *c* (Å)	12.966 (2), 13.339 (4)	12.8634 (4), 13.2923 (9)
V ($Å^3$)	2242.4 (10)	2199.4 (2)
μ (mm^{-1})	2.37	2.41
Crystal size (mm)	0.10 × 0.08 × 0.05	0.10 × 0.08 × 0.08
Data collection		
Diffractometer	Bruker Kappa Apex2	Bruker Kappa Apex2
T_{min}, T_{max}	0.22, 0.89	0.60, 0.82
No. of measured, independent and observed reflections	1978, 1050, 745 [$I > 2.0\sigma(I)$]	3596, 1089, 943 [$I > 2.0\sigma(I)$]
R_{int}	0.070	0.038
θ_{max} (°)	23.4	24.7
$(\sin\theta/\lambda)_{max}$ ($Å^{-1}$)	0.558	0.589
Refinement		
$R[F^2 > 2\sigma(F^2)]$, $wR(F^2)$, S	0.069, 0.059, 0.99	0.054, 0.029, 0.95
No. of reflections	745	943
No. of parameters	139	139
No. of restraints	84	84
H-atom treatment	H-atom parameters constrained	H-atom parameters constrained
$\Delta\rho_{max}$, $\Delta\rho_{min}$ (e $Å^{-3}$)	0.26, -0.23	0.19, -0.14
Absolute structure	Flack (1983), 482 Friedel pairs	Flack (1983), 568 Friedel pairs
Absolute structure parameter	0.51 (9)	0.44 (3)

Supplementary Table 9: QTAIM metrics for all $M(OAr)_4$ systems. $\rho_{BCP(M,O)}$, $-(G/V)_{BCP}$ and δ(M,O) are given in atomic units (a.u.).

$Th(OAr)_4$

Pressure, GPa	*r*(M-O), Å	Error	Δ *r*(M,O), Å	Δ *E*, $kJ \cdot mol^{-1}$	$\rho_{BCP(M,O)}$	$-(G/V)_{BCP}$	δ(M,O)
Ambient	2.1861	0.0017	0	0.0	0.102	0.787	0.666
0.26	2.184	0.004	-0.002	11.8	0.102	0.786	0.668
0.52	2.173	0.003	-0.013	13.4	0.104	0.783	0.674
0.58	2.176	0.006	-0.010	14.4	0.104	0.783	0.673
0.91	2.164	0.004	-0.022	35.5	0.106	0.780	0.682
1.37	2.153	0.004	-0.033	44.0	0.109	0.773	0.698
1.77	2.159	0.004	-0.027	56.2	0.108	0.774	0.695
2.04	2.156	0.004	-0.030	54.3	0.109	0.772	0.699
2.41	2.157	0.004	-0.029	60.7	0.109	0.769	0.700
2.44	2.178	0.003	-0.008	58.8	0.105	0.777	0.685
2.62	2.180	0.003	-0.006	53.6	0.104	0.776	0.682
2.83	2.188	0.005	0.002	78.1	0.103	0.778	0.676
2.88	2.175	0.003	-0.011	56.8	0.106	0.774	0.685
3.03	2.074	0.011	-0.112	187.8	0.129	0.746	0.758
3.16	2.132	0.016	-0.054	122.9	0.115	0.757	0.717
3.42	2.117	0.010	-0.069	164.0	0.120	0.750	0.728
4.30	2.055	0.019	-0.131	212.9	0.136	0.729	0.771

$U(OAr)_4$

Pressure, GPa	*r*(M-O), Å	Error	Δ *r*(M,O), Å	Δ *E*, $kJ \cdot mol^{-1}$	$\rho_{BCP(M,O)}$	$-(G/V)_{BCP}$	δ(M,O)
Ambient	2.1444	0.0019	0.000	0.0	0.108	0.797	0.734
0.14	2.138	0.006	-0.006	11.3	0.110	0.794	0.738
0.30	2.131	0.007	-0.013	12.0	0.111	0.791	0.746
0.44	2.132	0.004	-0.012	30.1	0.111	0.792	0.745
0.56	2.123	0.003	-0.021	17.3	0.113	0.790	0.750
0.70	2.129	0.007	-0.015	13.7	0.112	0.792	0.745
1.11	2.124	0.005	-0.020	37.0	0.113	0.788	0.756
1.84	2.115	0.006	-0.029	63.7	0.116	0.782	0.769
2.37	2.114	0.005	-0.030	65.8	0.117	0.780	0.771
2.47	2.115	0.005	-0.029	61.4	0.116	0.781	0.771
2.67	2.114	0.004	-0.030	62.7	0.117	0.779	0.772
2.67	2.110	0.006	-0.034	99.7	0.117	0.780	0.774
2.85	2.117	0.005	-0.027	71.5	0.116	0.781	0.768
2.88	2.124	0.005	-0.020	83.2	0.114	0.783	0.761
3.02	2.073	0.012	-0.071	110.8	0.126	0.773	0.800
3.32	2.089	0.011	-0.055	149.3	0.130	0.758	0.808
3.88	2.077	0.013	-0.067	147.4	0.127	0.762	0.802

$Np(OAr)_4$

Pressure, GPa	*r*(M-O), Å	Error	Δ *r*(M,O), Å	Δ *E*, $kJ \cdot mol^{-1}$	$\rho_{BCP(M,O)}$	$-(G/V)_{BCP}$	δ(M,O)
Ambient	2.119	0.003	0.000	0.0	0.114	0.791	0.769
0.54	2.111	0.100	-0.008	25.7	0.116	0.787	0.781
1.00	2.110	0.090	-0.009	40.2	0.116	0.786	0.782
1.55	2.108	0.011	-0.011	55.7	0.120	0.783	0.789
2.04	2.075	0.015	-0.044	64.1	0.130	0.772	0.823
2.38	2.054	0.008	-0.065	99.0	0.137	0.763	0.842

Supplementary Table 10: NLMO metrics for all M(OAr)$_4$ systems.

**Th(OAr)$_4$**	**σ:**						**π (average):**					
**Pressure, GPa**	**%M**	**s**	**p**	**d**	**f**	**Overlap**	**%M**	**s**	**p**	**d**	**f**	**Overlap**
Ambient	6.19	7.8	0.6	69.0	22.5	0.390	4.73	0.1	0.2	57.2	42.3	0.425
0.26	6.11	7.5	0.6	69.5	22.3	0.383	4.69	0.1	0.2	57.5	42.1	0.423
0.52	6.13	7.3	0.6	69.8	22.2	0.374	4.68	0.1	0.2	57.4	42.2	0.423
0.58	6.11	7.2	0.6	69.9	22.2	0.379	4.69	0.1	0.2	57.5	42.1	0.424
0.91	6.05	6.5	0.6	70.8	21.9	0.364	4.66	0.1	0.2	57.7	41.8	0.423
1.37	6.02	5.1	0.7	72.6	21.6	0.352	4.68	0.1	0.2	58.3	41.2	0.425
1.77	5.94	4.3	0.6	73.7	21.3	0.353	4.64	0.1	0.2	58.8	40.6	0.411
2.04	5.94	4.0	0.6	74.0	21.3	0.353	4.66	0.1	0.2	59.0	40.5	0.411
2.41	5.89	3.5	0.6	74.3	21.4	0.357	4.68	0.1	0.2	59.2	40.2	0.414
2.44	5.77	3.9	0.6	74.0	21.4	0.348	4.66	0.1	0.2	59.4	40.0	0.424
2.62	5.78	4.1	0.5	73.8	21.5	0.350	4.65	0.1	0.2	59.3	40.1	0.422
2.83	5.69	3.8	0.5	74.0	21.5	0.348	4.65	0.1	0.3	59.7	39.8	0.419
2.88	5.81	4.0	0.6	73.7	21.6	0.350	4.69	0.1	0.2	59.3	40.1	0.423
3.03	6.31	1.8	0.8	76.5	20.7	0.318	4.72	0.1	0.2	60.4	39.0	0.430
3.16	5.95	3.2	0.8	74.7	21.1	0.347	4.54	0.1	0.3	59.3	40.1	0.395
3.42	6.02	2.5	0.8	75.4	21.1	0.346	4.53	0.1	0.3	59.7	39.7	0.390
4.30	6.43	2.7	1.1	74.6	21.5	0.300	4.60	0.2	0.4	58.8	40.4	0.411
**U(OAr)$_4$**	**σ:**						**π (average):**					
**Pressure, GPa**	**%M**	**s**	**p**	**d**	**f**	**Overlap**	**%M**	**s**	**p**	**d**	**f**	**Overlap**
Ambient	6.55	6.2	0.5	58.6	34.7	0.389	6.30	0.1	0.2	44.3	55.4	0.437
0.14	6.50	5.7	0.5	59.0	34.8	0.385	6.24	0.1	0.2	44.4	55.3	0.434
0.30	6.48	5.3	0.6	59.3	34.8	0.383	6.28	0.1	0.2	44.4	55.3	0.436
0.44	6.40	5.2	0.6	61.0	33.2	0.368	6.30	0.1	0.2	43.9	55.8	0.439
0.56	6.63	5.2	0.6	59.1	35.2	0.381	6.28	0.1	0.2	44.4	55.3	0.439
0.70	6.62	5.0	0.6	58.0	36.5	0.382	6.21	0.1	0.2	44.5	55.2	0.434
1.11	6.39	4.0	0.7	62.0	33.3	0.366	6.26	0.1	0.3	44.4	55.2	0.425
1.84	6.55	2.8	0.7	65.4	31.1	0.374	6.51	0.1	0.3	44.5	55.1	0.426
2.37	6.72	2.5	0.5	61.9	35.1	0.389	6.43	0.1	0.2	46.3	53.3	0.432
2.47	6.70	2.4	0.5	62.0	35.1	0.390	6.45	0.1	0.2	46.5	53.1	0.433
2.67	6.68	2.4	0.5	62.0	35.1	0.392	6.46	0.1	0.2	46.5	53.1	0.431
2.67	6.65	2.7	0.7	64.7	31.9	0.386	6.74	0.1	0.2	44.3	55.4	0.456
2.85	6.49	2.6	0.6	65.2	31.7	0.382	6.58	0.1	0.2	43.9	55.8	0.432
2.88	6.59	2.5	0.5	61.7	35.3	0.390	6.40	0.1	0.2	46.1	53.5	0.426
3.02	7.40	2.0	0.5	62.0	35.5	0.389	6.63	0.1	0.1	45.8	54.0	0.448
3.32	6.41	1.2	0.6	62.5	34.9	0.380	6.49	0.1	0.3	45.4	54.2	0.438
3.88	6.96	1.7	0.7	63.1	34.5	0.378	6.37	0.1	0.2	47.2	52.5	0.438
**Np(OAr)$_4$**	**σ:**						**π (average):**					
**Pressure, GPa**	**%M**	**s**	**p**	**d**	**f**	**Overlap**	**%M**	**s**	**p**	**d**	**f**	**Overlap**
Ambient	6.24	6.5	0.8	59.6	33.1	0.345	7.39	0.0	0.2	36.8	63.0	0.414
0.54	6.05	5.5	0.9	59.9	33.7	0.332	7.38	0.0	0.3	36.9	62.8	0.413
1.00	5.98	5.1	0.9	60.0	34.1	0.323	7.34	0.1	0.3	36.2	63.0	0.410
1.55	5.99	3.9	0.9	61.6	33.6	0.327	7.42	0.1	0.3	37.4	62.2	0.407
2.04	6.34	2.9	1.0	62.6	33.5	0.328	7.50	0.1	0.3	37.8	61.8	0.417
2.38	6.65	2.8	1.0	62.1	34.0	0.332	7.60	0.1	0.3	37.9	61.8	0.423

3. Supplementary Figures

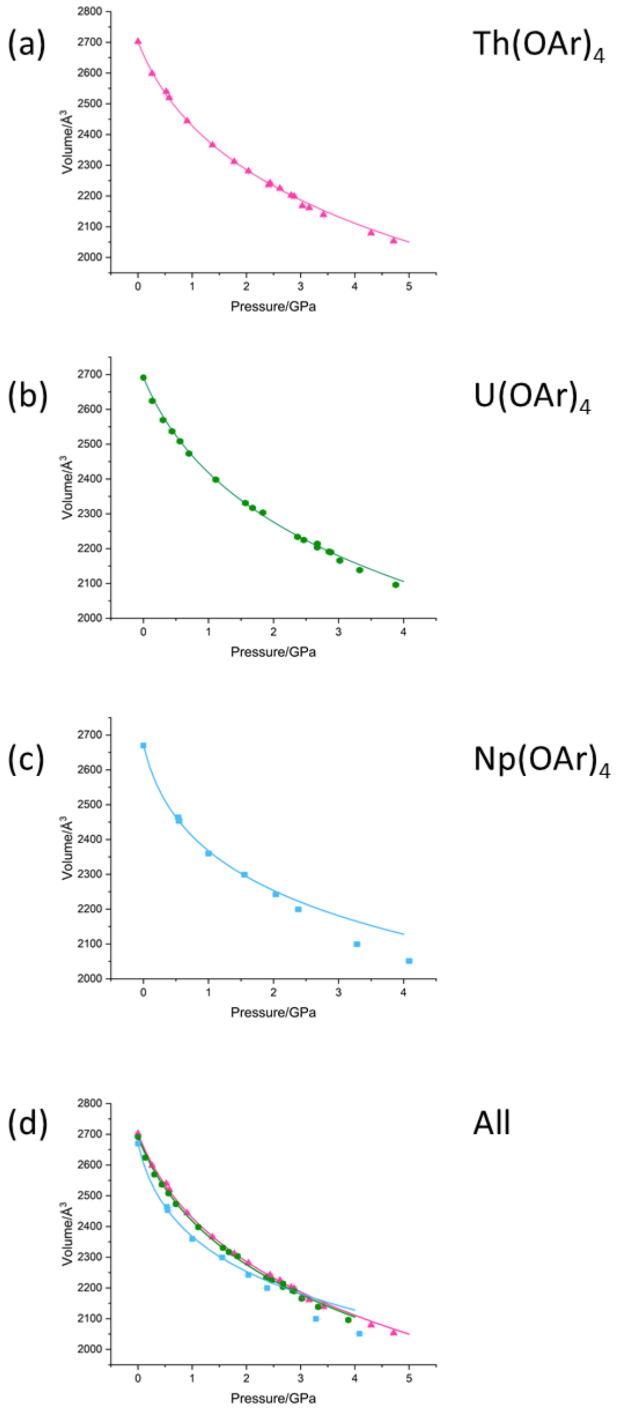

Supplementary Figure 7: Variation of unit cell volume (Å^3) with pressure (GPa) at ambient temperature for M(OAr)$_4$ where M = (a) Th, (b) U, (c) Np. In (d) all data are plotted on a common set of axes. The pressure points are fitted to Vinet equation of states using the points to 2.6 GPa for M = Th and U and 1.5 GPa for M = Np. Note that the points fall below the extrapolated equation of state curves at 2.88 and 3.02 GPa for M = Th and U and 2.3 GPa for M = Np. EoS parameters (V_0/Å^3, K_0/GPa and K'_0) are: 2702.29(9), 6.0(4) and 8.4(7) for M = Th; 2691.17(17), 6.0(3), and 8.5(6) for M = U and 2669.9(15), 3.5(5) and 15(2) for M = Np. The limited range of data available and resulting parameter correlation is likely to be responsible for the apparently very different values for M = Np.

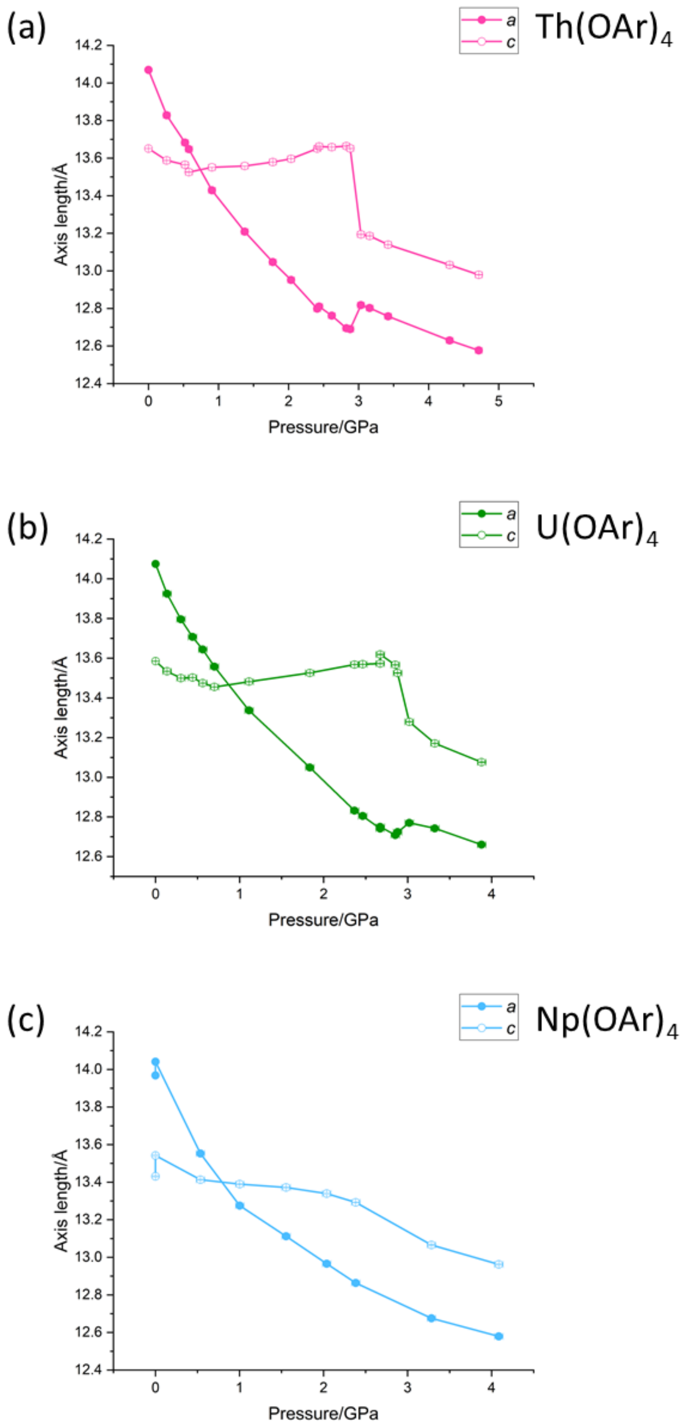

Supplementary Figure 8: Variation of unit cell axis lengths (Å) with pressure (GPa) at ambient temperature for $M(OAr)_4$ where M = (a) Th, (b) U, (c) Np. The last data point in (a) and the two last data points in (c) yielded diffraction data suitable for determination of unit cell dimensions but not structure analysis. The upper and lower points in (c) at 0 GPa were collected at 253 and 100 K, respectively. All other points were collected at room temperature.

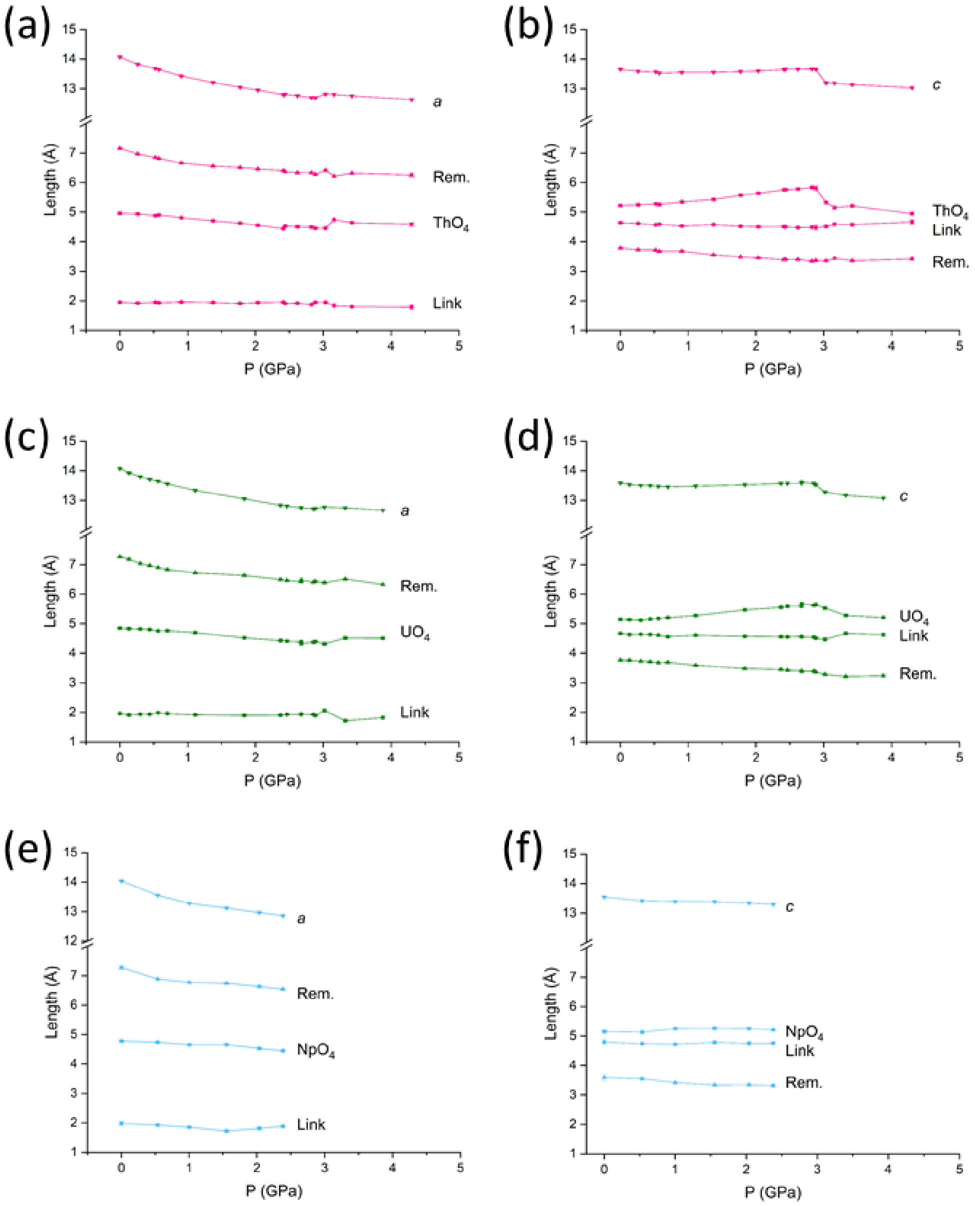

Supplementary Figure 9. A more detailed projection analysis for $Th(OAr)_4$, $U(OAr)_4$ and $Np(OAr)_4$. The plots show the contributions of the dimensions of the MO_4 tetrahedra (ThO_4 *etc.*), the 'link' region spanned by the ligating oxygen atom ('Link') and the remainder of the structure (Rem.) to the *a* and *c* unit cell dimensions. Details of the calculations are given in Section 1.11. (a) and (b) show the analysis for $Th(OAr)_4$ along *a* and *c*, respectively; (c) and (d) show the same analyses for $U(OAr)_4$, and (e) and (f) show the analyses for $Np(OAr)_4$. The Figure shows that the changes in the MO_4 moieties are primarily responsible for the changes in the *a* and *c* unit cell dimensions at the phase transitions.

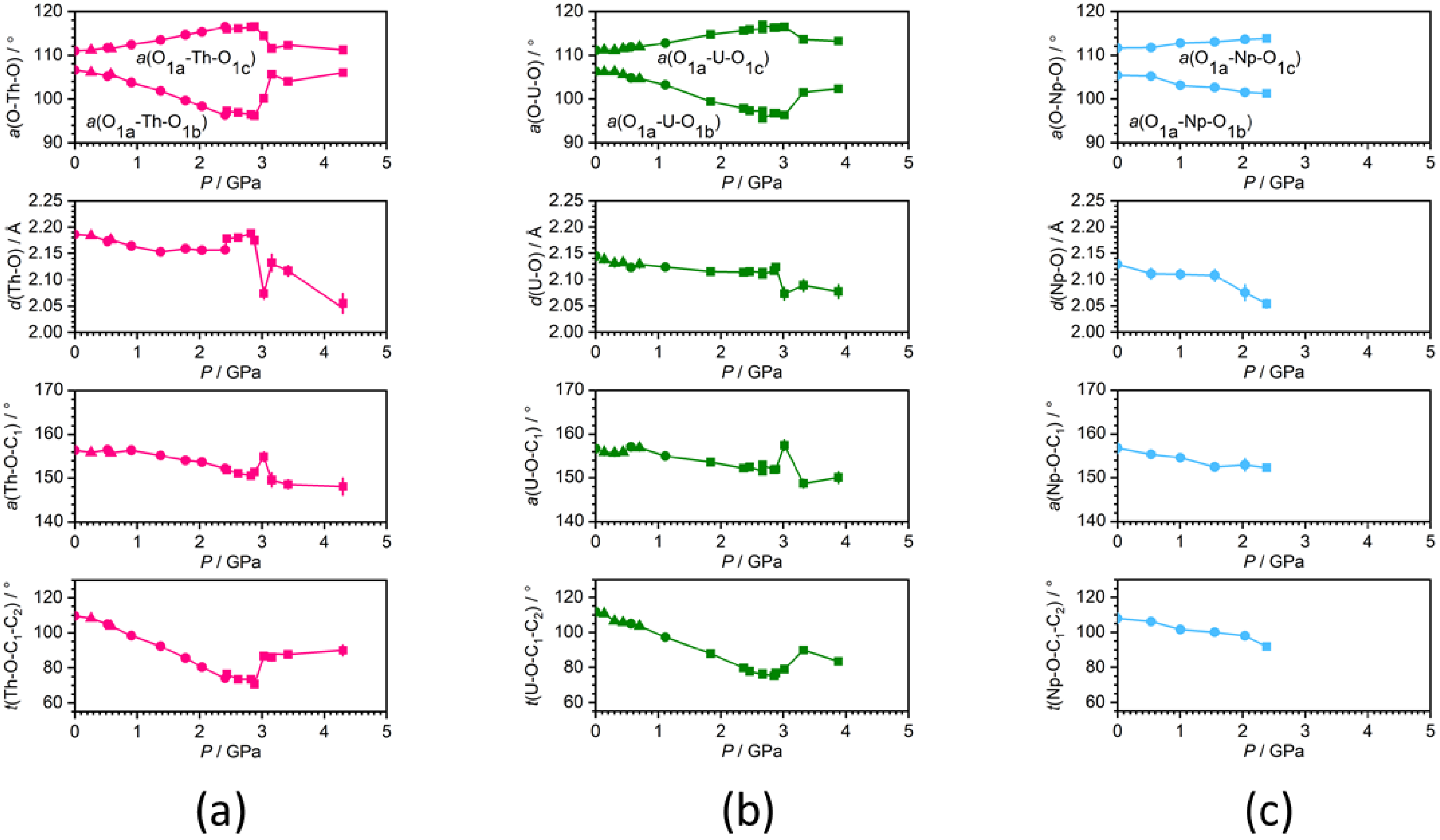

Supplementary Figure 10. The influence of pressure on the structural parameters of M(OAr)$_4$ Where M = Th, U and Np in (a), (b) and (c), respectively. This Figure complements Fig 4 in the main text. Data obtained with hydrostatic media Fluorinert FC70, Daphne 7373 and 1:1 pentane and isopentane are shown as triangles, circles and squares, respectively. The panels depict from top to bottom: the two unique O-M-O angles of the MO$_4$ unit (see Fig. 1 of the main text for labels); the M-O distance; the angle subtended at oxygen, M-O-C$_1$; the dihedral angle describing the tilt of the phenol rings: t(M-O-C$_1$-C$_2$). Error bars taken from the crystallographic structure refinements are plotted, but are mostly smaller than symbols used for the data points.

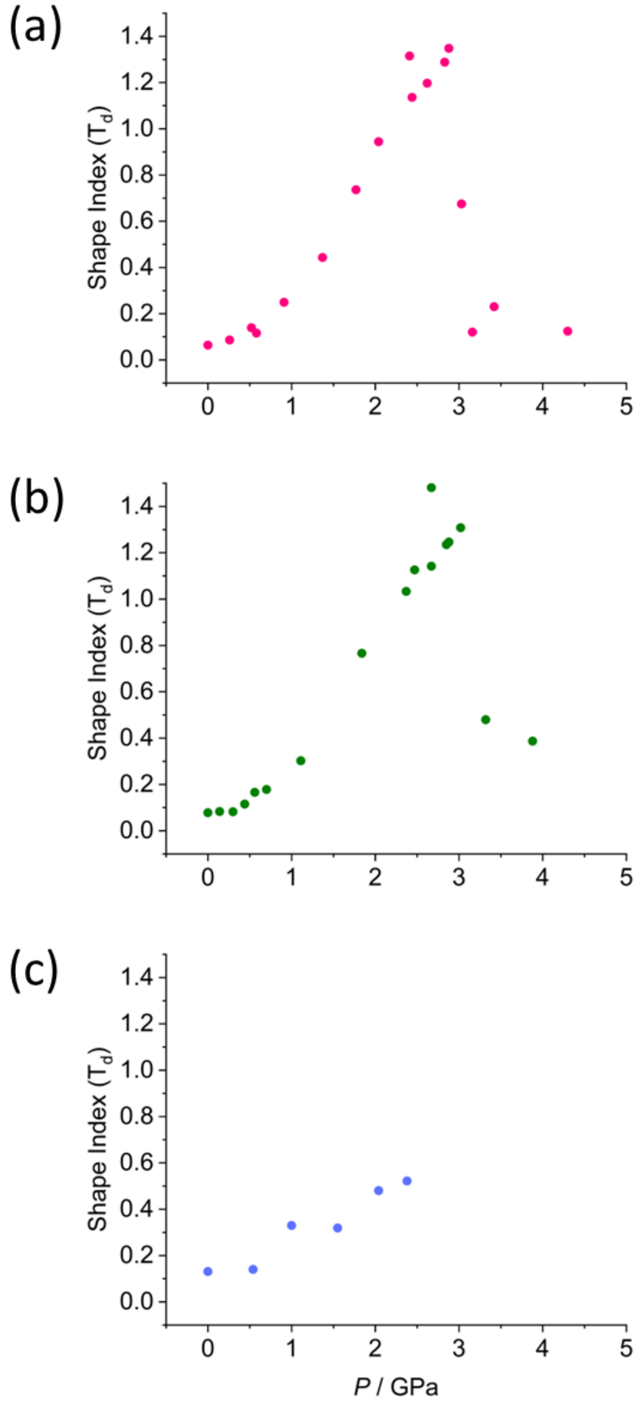

Supplementary Figure 11. Shape indices relative to T_d point symmetry for the MO_4 moieties in $M(OAr)_4$ as a function of pressure. (a) M = Th, (b) M = U, (c) M = Np. All complexes have crystallographic S_4 (or -4) symmetry and exact T_d symmetry would yield a shape index of zero. Details of the calculation are given in Section 1.18 of this document.

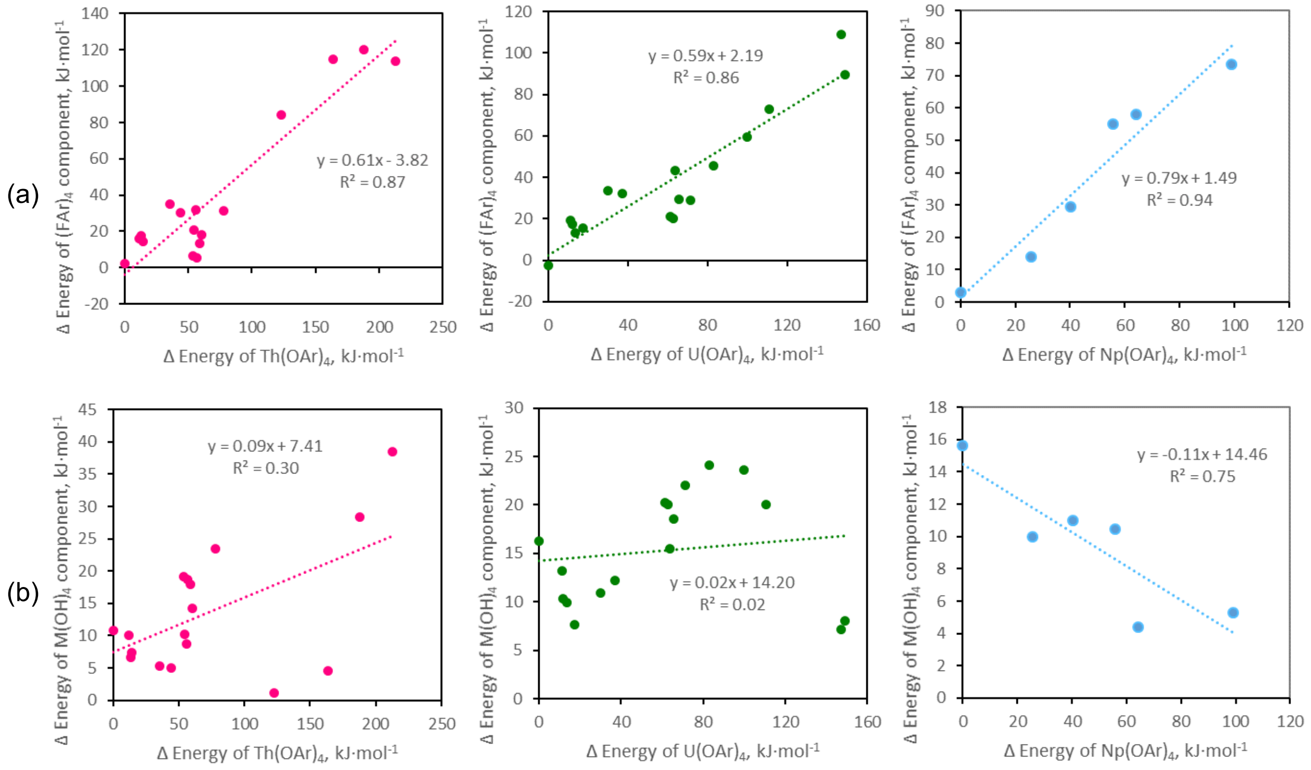

Supplementary Figure 12: Correlation of the total $M(OAr)_4$ energy via estimates with the (a) ligand repulsion energies and (b) core energies. The panels depict from left to right data for $Th(OAr)_4$, $U(OAr)_4$ and ($Np(OAr)_4$. Ligand repulsion energies are calculated from the calculation of the fluorine substituted ligands, in the absence of the central actinide. The core energies are from the calculation of the $M(OH)_4$ system, where the actinide and oxygen positions are fixed as per the experimental structures.

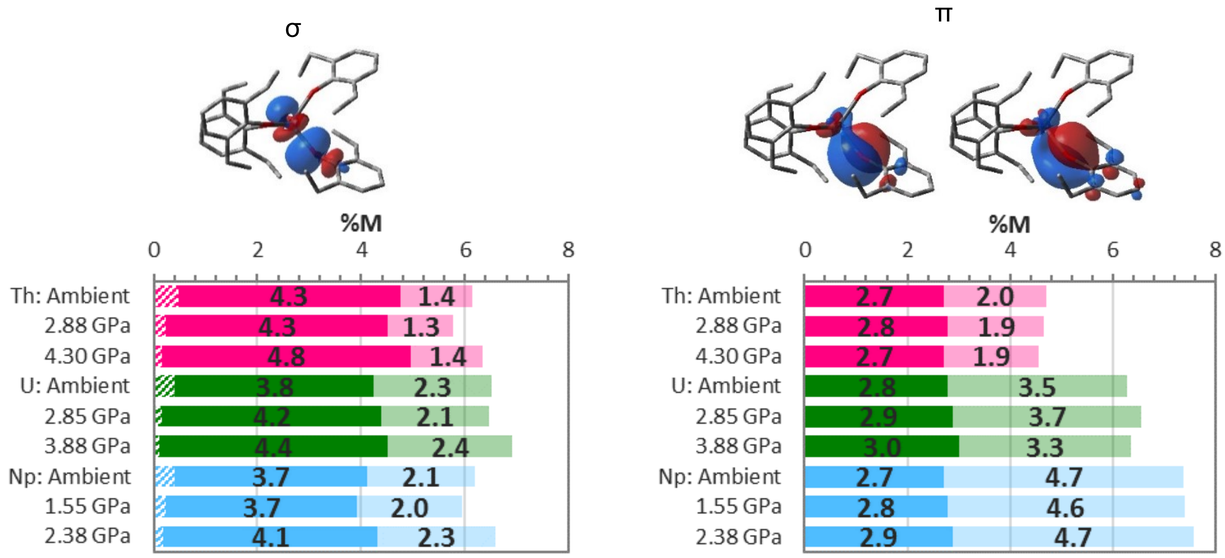

Supplementary Figure 13: Metal contribution (%M) to M-O bonding NLMOs are decomposed in the σ-type M-O bonding NLMOs (left) and averaged π-type bonding (right). Relative contributions from s (lined), d (dark), and f (light) orbitals are represented. There is no significant (>0.1%) p-orbital contributions.

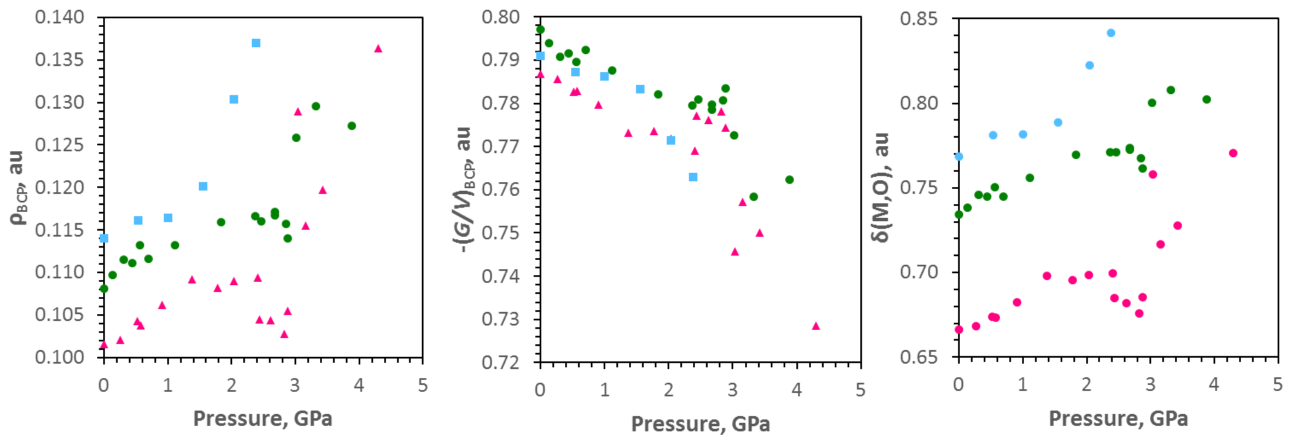

(a) (b) (c)

Supplementary Figure 14: QTAIM metrics for $M(OAr)_4$ as a function of pressure: electron density at the M-O bond critical points [ρ_{BCP}] shown in (a); the ratio of kinetic and potential energy densities at the M-O bond critical point [– $(G/V)_{BCP}$] shown in (b); and delocalization index [δ(M-O)] shown in (c). All metrics in atomic units (au).

4. Supplementary References

1. Geerts RL, Huffman JC, Caulton KG. Soluble zinc bis(aryloxides). *Inorg Chem* **25**, 1803-1805 (1986).

2. Schneider D, Spallek T, Maichle-Mössmer C, Törnroos KW, Anwander R. Cerium tetrakis(diisopropylamide) – a useful precursor for cerium(iv) chemistry. *Chem Comm* **50**, 14763-14766 (2014).

3. Dormond A, El Bouadili A, Aaliti A, Moise C. Insertion of carbonyl compounds into actinide—carbon σ bonds: Reactivity of $[((Me_3Si)_2)_2N]_2M.CH_2Si(Me)_2NSiMe_3$. *J Organometallic Chem* **288**, C1-C5 (1985).

4. Cantat T, Scott BL, Kiplinger JL. Convenient access to the anhydrous thorium tetrachloride complexes $ThCl_4(DME)_2$, $ThCl_4(1,4\text{-dioxane})_2$ and $ThCl_4(THF)_{3.5}$ using commercially available and inexpensive starting materials. *Chem Comm* **46**, 919-921 (2010).

5. Covert KJ, Mayol A-R, Wolczanski PT. Carbon-oxygen and related RX bond cleavages mediated by (silox)3Ti and other Group 4 derivatives (silox = tBu3SiO). *Inorg Chim Acta* **263**, 263-278 (1997).

6. Laubereau PG. Präparative und radiochemische Synthesen von Cyclopentadienylkomplexen der Actiniden und des Promethiums sowie Untersuchungen zum Spaltprodukteinbau in Aromaten-Fängerkomplexe. (ed^(eds). Technische Hochschule München (1966).

7. Choporov DY, Chudinov EG. Melting point and saturated vapor pressure of neptunium tetrachloride. *Radiokhimiya* **10**, 221-227 (1968).

8. Berg JM*, et al.* Early actinide alkoxide chemistry. Synthesis, characterization, and molecular structures of thorium(IV) and uranium(IV) aryloxide complexes. *J Am Chem Soc* **114**, 10811-10821 (1992).

9. Yin H, Carroll PJ, Manor BC, Anna JM, Schelter EJ. Cerium Photosensitizers: Structure–Function Relationships and Applications in Photocatalytic Aryl Coupling Reactions. *J Am Chem Soc* **138**, 5984-5993 (2016).

10. García-Álvarez J, Graham DV, Hevia E, Kennedy AR, Mulvey RE. Synthesis and characterisation of new bimetallic alkali metal–magnesium mixed diisopropylamide-acetylides: structural variations in bimetallic lithium- and sodium-heteroleptic magnesiates. *Dalton Trans*, 1481-1486 (2008).

11. Arnold PL, Casely IJ, Zlatogorsky S, Wilson C. Organometallic Cerium Complexes from Tetravalent Coordination Complexes. *Helv Chim Acta* **92**, 2291-2303 (2009).

12. Falivene L*, et al.* SambVca 2. A Web Tool for Analyzing Catalytic Pockets with Topographic Steric Maps. *Organometallics* **35**, 2286-2293 (2016).

13. Van Der Sluys WG, Sattelberger AP, Streib WE, Huffman JC. Tetrakis(2,6-di-t-butylphenoxy)uranium(IV): The first structurally characterized neutral homoleptic aryloxide complex of uranium(IV). *Polyhedron* **8**, 1247-1249 (1989).

14. Haynes WM. *CRC Handbook of Chemistry and Physics*. CRC Press (Taylor and Francis Group LLC, Boca Raton) (2017).

15. Lateskey S, Keddington J, McMullen AK, Rothwell IP, Huffman JC. Chemistry of sterically crowded aryloxide ligands. 5. Synthesis, structure, spectroscopic properties, and electrochemical behavior of group 4 metal derivatives containing bulky aryloxide ligands. *Inorg Chem* **24**, 995-1001 (1985).

16. Merrill L, Bassett WA. Miniature diamond anvil pressure cell for single crystal x‐ray diffraction studies. *Rev Sci Inst* **45**, 290-294 (1974).

17. Moggach SA, Allan DR, Parsons S, Warren JE. Incorporation of a new design of backing seat and anvil in a Merrill-Bassett diamond anvil cell. *J Appl Cryst* **41**, 249-251 (2008).

18. Sheldrick G. SHELXT - Integrated space-group and crystal-structure determination. *Acta Cryst* **A71**, 3-8 (2015).

19. Angel RJ, Alvaro M, Gonzalez-Platas J. EosFit7c and a Fortran module (library) for equation of state calculations. *ZKrist* **229**, 405-419 (2014).

20. Pinsky M, Avnir D. Continuous Symmetry Measures. 5. The Classical Polyhedra. *Inorg Chem* **37**, 5575-5582 (1998).

21. Alvarez S, Alemany P, Casanova D, Cirera J, Llunell M, Avnir D. Shape maps and polyhedral interconversion paths in transition metal chemistry. *Coord Chem Rev* **249**, 1693-1708 (2005).

22. Llunell M, Casanova D, Cirera J, Alemany P, Alvarez S. SHAPE. Version 2.1 edn (2013).
