# Supplementary material for: Covalent bond shortening and distortion induced by pressurization of thorium, uranium, and neptunium tetrakis aryloxides
Source: Nat Commun. 2022 Oct 7;13:5923. doi: 10.1038/s41467-022-33459-7 (PMC9546877; doi:10.1038/s41467-022-33459-7)
Supplement: Supplementary file 2 — Description of Additional Supplementary Files [file 41467_2022_33459_MOESM2_ESM.pdf]

Supplementary Movie 1. Compression of $Th(OAr)_4$ viewed along the *b* axis showing rotation of the tBu groups with pressure. H-atoms omitted for clarity. The origin has been shifted in this and other plots to place the metal atoms at (000) and (½½½), with the layer at *y* = ½ shown.

Supplementary Movie 2. Compression of $Th(OAr)_4$ viewed along the *c* axis showing rotation of the molecules with pressure. H-atoms omitted for clarity. The layer at *z* = ½ is shown.

Supplementary Movie 3. Compression of $Th(OAr)_4$ viewed along the *c* axis showing development of large thermal ellipsoids and disorder in the tBu groups as they rotate with increasing pressure. H-atoms omitted for clarity.

Supplementary Movie 4. Compression of $U(OAr)_4$ viewed along the *b* axis showing rotation of the tBu groups with pressure. H-atoms omitted for clarity.

Supplementary Movie 5. Compression of $U(OAr)_4$ viewed along the *c* axis showing rotation of the molecules with pressure. H-atoms omitted for clarity.

Supplementary Movie 6. Compression of $U(OAr)_4$ viewed along the *c* axis showing development of large thermal ellipsoids and disorder in the tBu groups as they rotate with increasing pressure. H-atoms omitted for clarity.

Supplementary Movie 7. Compression of $Np(OAr)_4$ viewed along the *b* axis showing rotation of the tBu groups with pressure. H-atoms omitted for clarity.

Supplementary Movie 8. Compression of $Np(OAr)_4$ viewed along the *c* axis showing rotation of the molecules with pressure. H-atoms omitted for clarity.

Supplementary Movie 9. Compression of $Np(OAr)_4$ viewed along the *c* axis showing development of large thermal ellipsoids and disorder in the tBu groups as they rotate with increasing pressure. H-atoms omitted for clarity.
